# Spontaneous Brain Activity Emerges from Pairwise Interactions in the Larval Zebrafish Brain

**DOI:** 10.1103/PhysRevX.14.031050

**Published:** 2024-09-23

**Authors:** Richard E. Rosch, Dominic R. W. Burrows, Christopher W. Lynn, Arian Ashourvan

**Affiliations:** Department of Basic and Clinical Neuroscience, Institute of Psychiatry, Psychology and Neuroscience, King’s College London, London, United Kingdom; Departments of Neurology and Pediatrics, Columbia University Irving Medical Center, New York City, New York, USA; Department of Imaging Neuroscience, University College London, London, United Kingdom; MRC Centre for Neurodevelopmental Disorders, King’s College London, London, United Kingdom and Department of Cognitive Science, University of California, San Diego, California, USA; Department of Physics, Quantitative Biology Institute, and Wu Tsai Institute, Yale University, New Haven, Connecticut, USA; Department of Psychology, University of Kansas, Lawrence, Kansas, USA

**Keywords:** Biological Physics, Complex Systems, Statistical Physics

## Abstract

Brain activity is characterized by brainwide spatiotemporal patterns that emerge from synapse-mediated interactions between individual neurons. Calcium imaging provides access to *in vivo* recordings of whole-brain activity at single-neuron resolution and, therefore, allows the study of how large-scale brain dynamics emerge from local activity. In this study, we use a statistical mechanics approach—the pairwise maximum entropy model—to infer microscopic network features from collective patterns of activity in the larval zebrafish brain and relate these features to the emergence of observed whole-brain dynamics. Our findings indicate that the pairwise interactions between neural populations and their intrinsic activity states are sufficient to explain observed whole-brain dynamics. In fact, the pairwise relationships between neuronal populations estimated with the maximum entropy model strongly correspond to observed structural connectivity patterns. Model simulations also demonstrated how tuning pairwise neuronal interactions drives transitions between observed physiological regimes and pathologically hyperexcitable whole-brain regimes. Finally, we use virtual resection to identify the brain structures that are important for maintaining the brain in a physiological dynamic regime. Together, our results indicate that whole-brain activity emerges from a complex dynamical system that transitions between basins of attraction whose strength and topology depend on the connectivity between brain areas.

## I Introduction

Macroscopic dynamics emerge out of interactions between system components at the microscale. In the brain, neuronal action potentials are caused by the interplay between ionic conductances and membrane channel proteins [1], while neuronal network dynamics emerge from the synaptic connections formed between constituent neurons and their intrinsic activity [2]. At the largest scales, fluctuations in whole-brain dynamics might be driven by a diversity of molecular and neuronal behaviors at fine-grained scales. However, as in many physical systems, much of this microscale complexity will have a negligible influence over the macroscale properties of the system and, therefore, may be ignored when considering macroscopic brain dynamics. Investigating which microscale properties constrain macroscale brain dynamics is fundamental to understanding brain function, as macroscopic network dynamics are strongly linked to behavior and cognition [3–6].

To bridge the gap between the microscale and macroscale, we require models that can reproduce observed microscopic details while making minimal assumptions about the rest of the system. In other words, we require the maximum entropy model (MEM) consistent with the observed microscopic properties [7]. Here, we seek to understand whether collective patterns of neural activity can be viewed as emerging from the network of pairwise correlations between neuronal populations. To answer this question, we employ the pairwise MEM, which is tuned to match the observed average activities and pairwise correlations in neural activity, but remains explicitly agnostic to higher-order relationships [8]. From these microscopic details, the resulting model predicts the distribution over macroscopic states of collective neural activity. In this way, the pairwise MEM allows us to understand whether and to what extent macroscopic properties can arise from simple pairwise relationships at the microscale. Furthermore, once a pairwise MEM has been constructed, it can be used to make direct analogies to statistical physics, by constructing an energy landscape that defines the attractor states of the collective neural dynamics [9].

Several studies have demonstrated the utility of maximum entropy approaches in explaining brain activity in small neuronal populations, recorded from salamander retina [8,10], rat prefrontal cortex [11], and cat parietal cortex [12]. However, it remains unclear whether such minimal-assumption approaches may also be applied to entire brain network dynamics. Recent advancements in human whole-brain neuroimaging and large-scale computational models have identified key relationships between microscale and macroscale dynamics in the brain—indicating roles for local inhibition in shaping macroscopic functional connectivity [13], neurotransmitter dynamics in driving emergent whole-brain states [14], and neuronal population parameter shifts in causing pathological macroscopic brain states during seizures [15,16]. However, data derived from such whole-brain imaging approaches are typically recorded at poor spatial resolutions, such that neuronal activity is coarse grained into units consisting of millions of neurons, and such recordings are insensitive to the heterogeneity of microscale activity [17]. Therefore, to accurately test whether MEM is a valid model of macroscopic brain dynamics, we require techniques that enable whole-brain recordings with microscopic resolution. Here, we take advantage of the larval zebrafish, which has an optically accessible nervous system enabling the recording of single-neuron activity across the entire brain [18], making it ideal for the interrogation of multiscale brain dynamics [19]. Interestingly, microscale neuronal connectivity has been shown to regulate global brain states [20,21], and when dysregulated, can drive macroscopic brain dysfunction [22]. Therefore, probing microscale-macroscale relationships through MEMs using the larval zebrafish can provide mechanistic insight into the origins of brainwide dynamics in health and disease.

Here, we estimate the pairwise interactions between neural populations at different scales to understand the role of microscopic neuronal dynamics in shaping functional and pathological macroscopic brain states. To do this, we studied resting state whole brain dynamics recorded from 11 zebrafish larvae (≈84 minutes of concatenated time series) [23]. We used spatial and functional clustering to identify functional populations of neurons at different scales whose pairwise interactions best explained macroscopic brain activity. Our results demonstrate that spontaneous patterns of whole brain activity at larger scales (i.e., 12 regions) can be modeled as a stochastic process dictated only by cluster activation propensity and intercluster connectivity patterns. Moreover, we show that the estimated pairwise (i.e., second-order) model parameters echo the structural connectivity that has been reported between macroscopic brain areas. By reconstructing the basins of attraction between different states in the model, we can explain the state transition patterns observed in macroscopic brain data, thus demonstrating how low-order interactions between coarse clusters drive macroscopic brain dynamics. Finally, we present a numerical simulation to demonstrate that tuning the temperature parameter of the model drives the dynamics from a critical to a hyper-synchronous regime, resembling the transition to pathological hypersynchronous activity seen, for example, in epileptic seizures, thus predicting emergent brain dysfunction from pairwise coupling changes at lower scales. Our results suggest that a simple brain model based on the probabilistic interactions between regions governed by the strength of the connectivity patterns can mechanistically explain and predict emergent functional and pathological activity across the whole brain.

## II Results

### A Dimensionality reduction and functional neuronal clusters

Dimensionality reduction on high-dimensional neuroimaging datasets is commonly performed before fitting to a pairwise maximum entropy model for several reasons (see, e.g., Refs. [8,24,25]). First, fitting MEMs to high-dimensional datasets can be computationally expensive, making dimensionality reduction a necessary first step. Second, dimensionality reduction helps to identify and preserve the most relevant features in the dataset, uncovering patterns and relationships in the data that might not be immediately apparent in high-dimensional spaces. In this way, dimensionality reduction can provide insights into the relevant scales in the system’s dynamics, leading to more interpretable models and results.

**Fig. 1:**
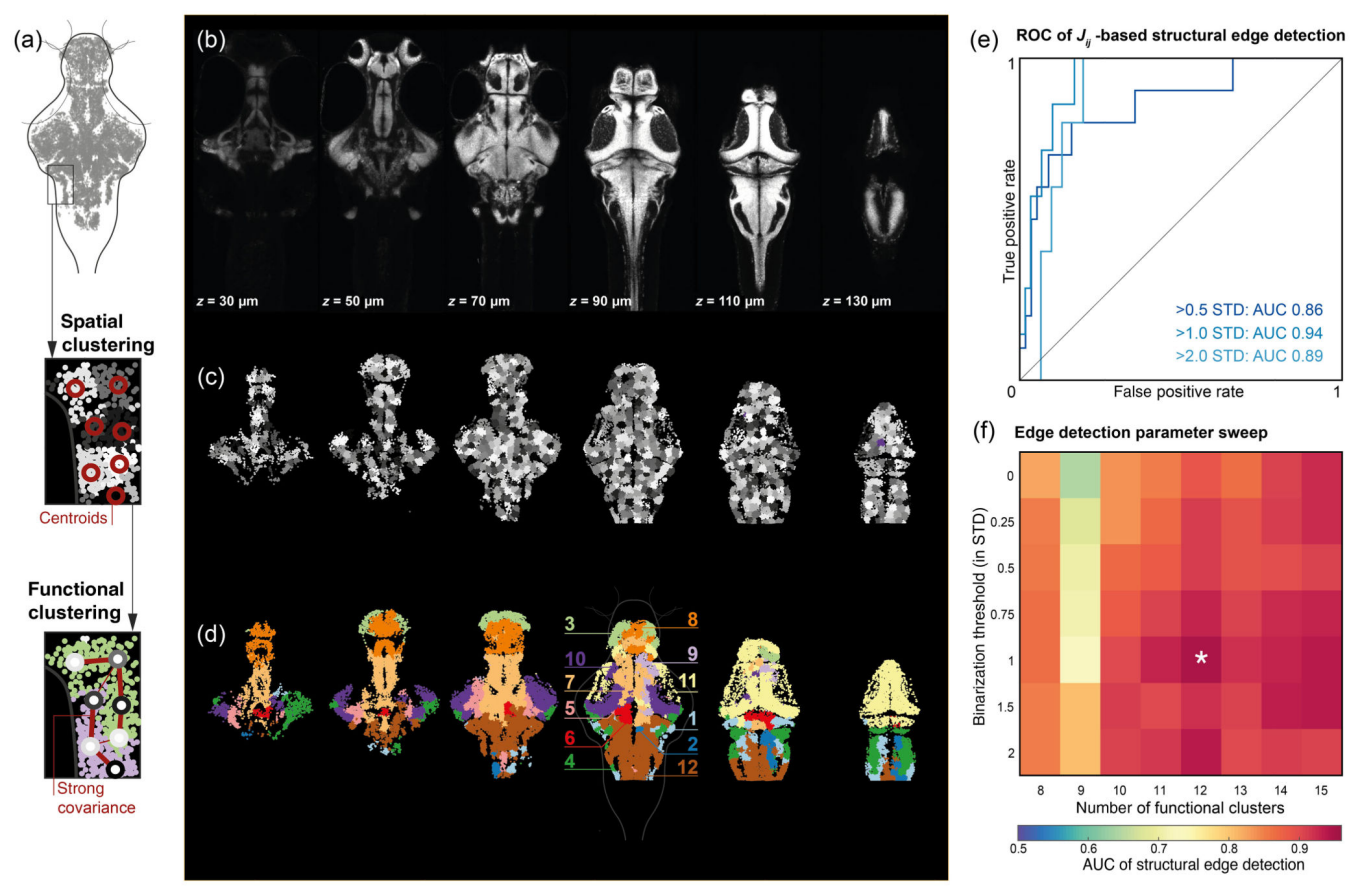
Coarse-grained functional clustering of individual neurons recorded across the zebrafish brain. (a) Workflow illustration: (top) neuron positions across multiple fish are coregistered to standard space, (middle) neurons are allocated to 1000 *k*-means clusters based on *xyz*-cell position in standard space, (bottom) spatial clusters of neurons are assigned a smaller number of functional clusters based on clustering of their covariance. (b) Horizontal sections through the zebrafish brain in the standard atlas space. (c) Spatial cluster assignments of individual neurons. (d) Functional cluster assignment of individual neurons. (e) AUC values for detection of structural edges based on *J*_*ij*_ matrix estimated based on clusters of different sizes and at different binarization thresholds of average cluster activations. The white asterisk marker shows the highest AUC value. (f) ROC for detecting structural edges (i.e., fiber count between clusters) for different structural edge thresholds [defined in relation to the standard deviation (STD) of all edges].

We identify the neurons that are functionally correlated and, therefore, may belong to a cluster via a two-step process. First, we realign all brains to the larval zebrafish Z-brain template atlas [26] and concatenate neurons from all larvae in three-dimensional space. Next, we used *k*-means clustering to identify 1000 anatomical regions of interest (ROIs) based on neurons’ spatial proximity [Fig. 1(a)]. Second, we used the covariance matrix of anatomical ROIs’ average calcium traces to identify the hierarchical functional clusters at different resolutions. We present the identified large-scale functional clusters for *N* = 12 in Fig. 1(d) and the number of cells per cluster distributions in Fig. 1 of Supplemental Material (SM) [27]. Additional clustering analyses reveal an optimal number of clusters at *N* = 2 and no clear optimal number at higher resolutions (for more details, see SM Fig. 2).

### B Pairwise MEM interaction weights echo the underlying structural connectivity

As outlined above, pairwise MEM is defined solely by the average activities of different brain regions and their functional correlations. From these microscopic details, the pairwise MEM then makes the maximally unbiased predictions for the probabilities of collective states of activity of the entire system without any additional constraints. Specifically, we treat the activity of each region as a binary variable, either “on” (*σ*_*i*_ = +1) or “off” (*σ*_*i*_ = −1), such that the collective activity of all regions is defined by the vector *σ*. We seek the distribution *P*(*σ*) over collective activity patterns that is consistent with the observed individual activation rates ⟨*σ*_*i*_⟩ and the pairwise correlations ⟨*σ*_*i*_*σ*_*j*_⟩, but otherwise has maximum entropy. This is the pairwise MEM:

(1) $$P(\sigma )=\frac{1}{Z}\text{exp}\left[\sum_{i=1}^{N}h_{i}\sigma_{i}+\frac{1}{2}\sum_{i\neq j}^{N}J_{ij}\sigma_{i}\sigma_{j}\right],$$

where *Z* is the normalizing partition function, and the parameters *h*_*i*_ and *J*_*ij*_ represent the activation biases of individual regions and the pairwise couplings between regions, respectively (see Sec. IV for more details). Importantly, the parameters *h*_*i*_ and *J*_*ij*_ are tuned such that the model matches the microscopic statistics ⟨*σ*_*i*_⟩ and ⟨*σ*_*i*_*σ*_*j*_⟩ observed in the real system.

We hypothesize that the estimated *J*_*ij*_, which captures the functional interactions between regions in the model, would show high similarity to the anatomical axonal (i.e., fiber count) connectivity between regions. As anticipated, a significant correlation was discovered between the estimated *J*_*ij*_ values and the strengths of structural connections (see SM Fig. 3 [27]). Likewise, the strength of the estimated functional interactions *J*_*ij*_ could reliably predict the presence of anatomical connectivity (i.e., binarized structural connectivity) between clusters, as indicated by high area under the curve (AUC) values of the receiver operating characteristic (ROC) curves [Fig. 1(e)]. We utilize the AUC of the ROC as a measure for the performance of binary classification of structural connectivity edges as present or absent based on *J*_*ij*_ edge weights across a range of binarization thresholds. Here, we show the binarization threshold (30% of maximum fiber count between clusters) that leads to the highest AUC values. We present similar results with different binarization thresholds in SM Fig. 4.

We can leverage this relationship between the inferred functional interactions *J*_*ij*_ and ground-truth structural connectivity to find the topological scale (i.e., clustering resolution) that maximizes this structure-function coupling. Although the small number of clusters (e.g., 5–7) yields the highest similarity between the structural connectivity and *J*_*ij*_ matrices, several clusters are spatially disjointed and scattered in both the anterior and posterior regions of the brain [see SM Fig. 3(e) [27]]. The next topological scale with high correlations (SM Fig. 3) and AUC values [Fig. 1(e)] is at *N* = 12 clusters (at the on state threshold of *z* = 1 for *z*-scored cluster activation time series), which will be used for the remainder of the analyses presented here. We also found converging results (see SM Figs. 3 and 5) for larger numbers of clusters (*N >* 15) with the MEM estimated using the pseudo-likelihood maximization method (see Sec. IV for more details).

In contrast to clustering with fewer but more distributed individual clusters, the *N* = 12 resolution does not exhibit anterior or posterior scattered clusters. Instead, the identified clusters are local and largely contiguous, as demonstrated in SM Fig. 3(e) [27]. Based on our hypothesis that the long-distance functional relationships between neuronal populations rely on the underlying anatomical connections between regions, we investigate the system’s behavior at *N* = 12 clusters [as depicted in Fig. 1(d)]. This topological scale maximizes the structure-function coupling and results in clusters characterized by local, contiguous populations scattered across the brain.

### C Pairwise MEM accurately predicts the probabilities of large-scale activation patterns

**Fig. 2:**
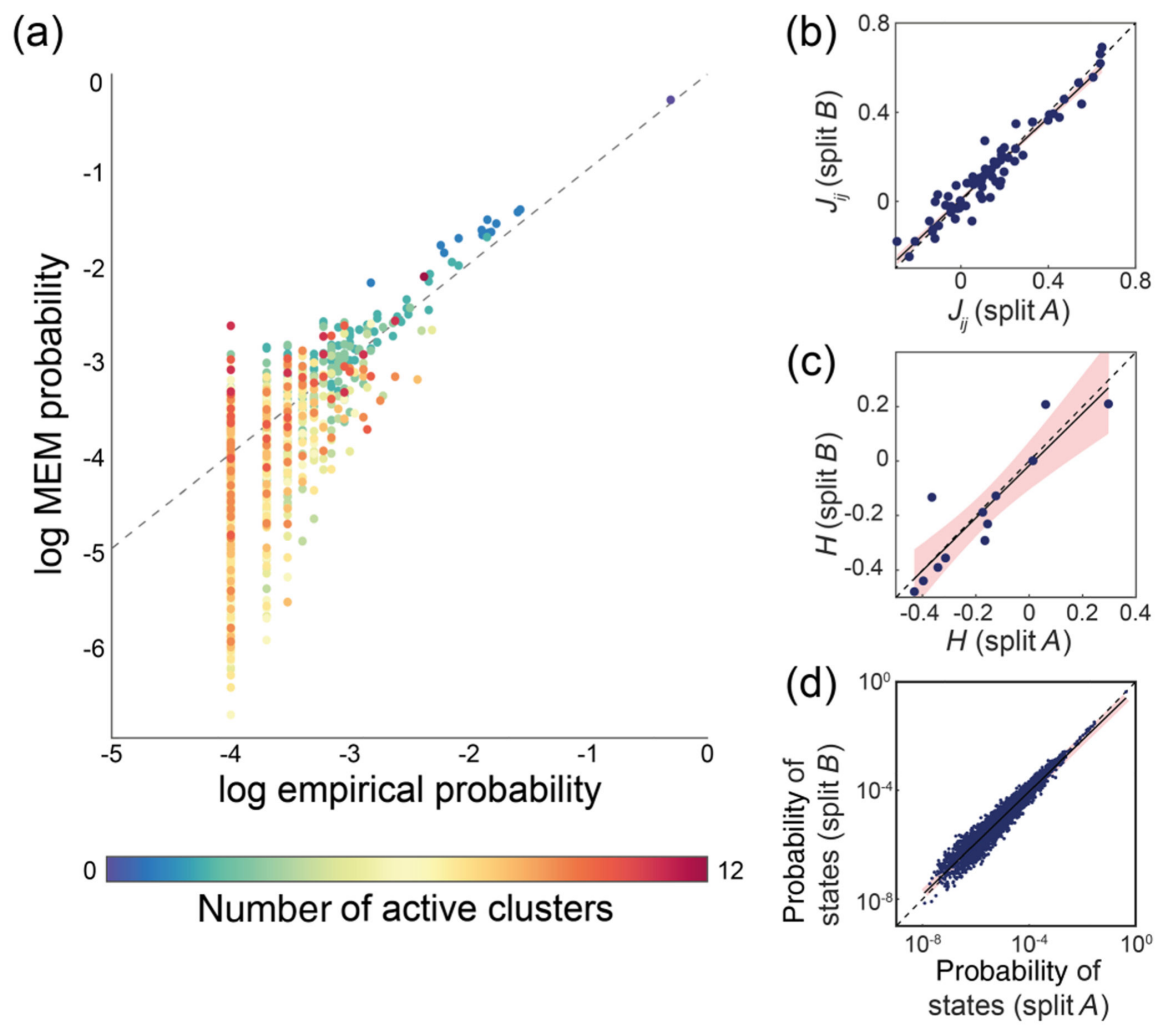
MEM predicts whole-brain activation patterns. (a) Pair-wise MEM-estimated and empirical probability of all states. Colors represent the number of active clusters. (b)–(d) Robustness of fit against noise within the data. Full datasets are randomly split into equally sized samples A and B. Correlation of sample A and B estimates of (b) *J*_*ij*_ (linear fit, *p* value ≈ 0, *R*^2^ = 0.95), (c) *h* (linear fit *p* value = 6.4 × 10^−6^, *R*^2^ = 0.81), and (d) the probabilities of all states (linear fit, *p* value ≈ 0, *R*^2^ = 0.93) are shown. Shaded areas show the confidence interval of the linear fit of the between-split correlation, which does not deviate significantly from the identity line.

We hypothesize that the spontaneous activity of largescale networks can be described as a stochastic process constrained by the activation rates of individual neuronal clusters and the strength of the functional connectivity between them. To test this hypothesis, we fit the pairwise MEM to the binarized patterns of collective cluster activity. See Sec. IV for details regarding the pairwise MEM model and parameter estimation. Our results demonstrate that the pairwise MEM accurately predicts the probabilities of commonly observed states [Fig. 2(a)]. Prediction accuracy is overall lower for states with low probability but is robust against inherent noise in the data [Figs. 2(b)–2(d)]. To model whole-brain state transitions with data that are feasible to record empirically, there is a trade-off: Recording length limits the amount of time the brain is captured, particularly in rare states, resulting in noisier estimates of state transition probabilities for high-dimensional state spaces.

We observe that the pairwise MEM offers a statistically supported model of observed activity, as it accounts for ≈84% of the multi-information that the first-order (i.e., independent) model does not capture. In SM Fig. 6, we show the goodness of fit of the model monotonically decreases by increasing the number of clusters [27]. Increasing the activation binarization threshold similarly lowers the goodness of fit at the smaller number of clusters. However, this relationship gradually reverses toward larger numbers of clusters (*N >* 13). In Sec. IV, we discuss the model’s goodness of fit in detail. Together, these results suggest that the pairwise MEM accurately describes the probability of the spontaneous large-scale activation patterns in the zebrafish brain.

### D Stochastic transitions between attractor basins of neural states

**Fig. 3:**
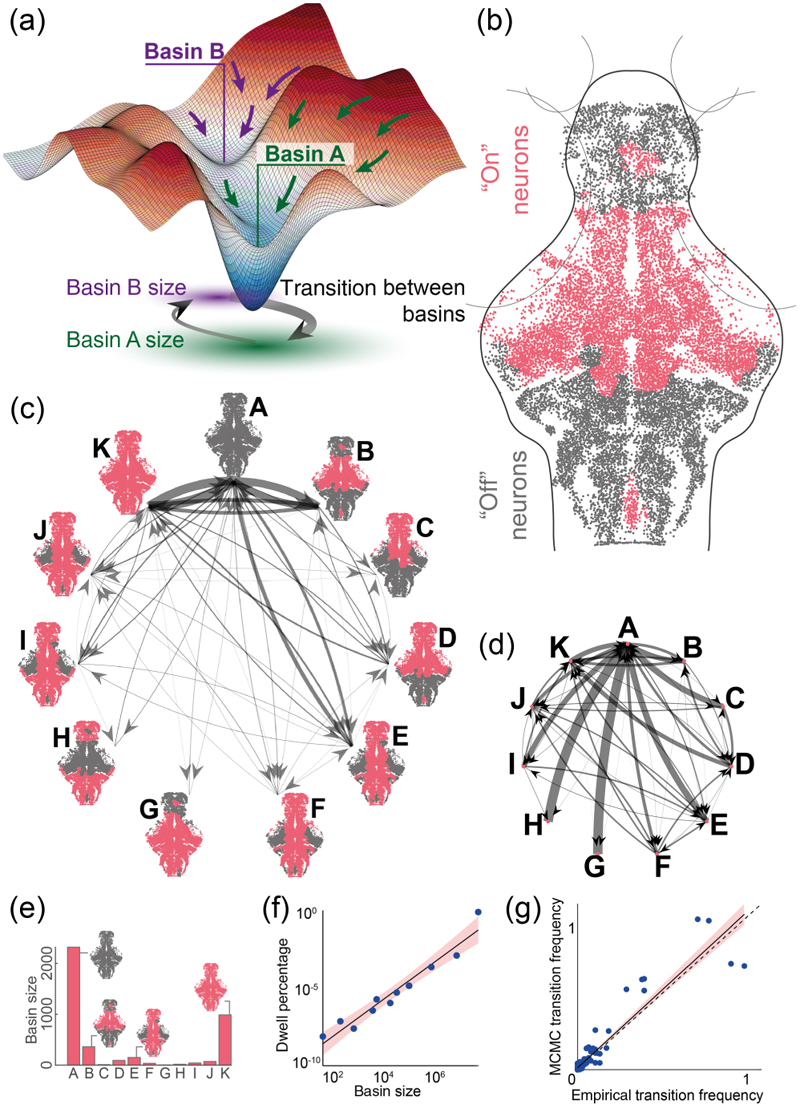
Attractor basins shape the energy landscape of whole-brain activation patterns. (a) Cartoon representation of high-dimensional energy landscape in which attractor states are defined as local minima, with surrounding basins consisting of brain states with transitions tending toward the attractor state. (b) Representative attractor state (B) consisting of on-off patterns across neuron clusters. (c) Empirically observed transition frequencies between 11 attractor states (A–K) identified for *N* = 12 clusters, with heterogeneous and asymmetric observed transition frequencies, with most frequent transitions from and to the “all-off” state A; thicker lines indicate a higher absolute number of transitions observed. (d) Empirical transition probabilities between the 11 attractor states in (c); thicker lines indicate higher transition probabilities from any one state, with the sum of all outgoing arrows being equal to 1. (e) Basin size for 11 attractor states. (f) Relationship between observed dwell times and the basin size of each attractor state. (g) Relationship between empirically observed transition frequencies and MCMC predicted transition frequencies based on the energy landscape.

Next, we set out to understand the constraints that define transitions between macroscopic brain states, particularly to understand if such transitions may be driven by a stochastic process over the energy landscape as defined by the MEM. Now that we have confirmed that the pairwise MEM accurately describes the large-scale neural patterns, we can use the model to explore the energy landscape over neural states. We first identified the attractor states of the estimated model by constructing a multidimensional mesh of all states, which we refer to as the energy landscape. In this landscape, neighboring activation states differ only in the activity of a single cluster. We exhaustively searched the energy landscape using a steep search algorithm to identify the states that are local maxima of the probability distribution (i.e., local minima of the energy landscape). The local minima were defined as states with higher probabilities than their neighboring states. To identify the states that belong to the basin of each local minimum, we found all neighboring states that transition on a downward gradient toward the minimum [Figs. 3(a) and 3(b); see Sec. IV].

In Fig. 3(c), we show the identified local minima for the *N* = 12 functional clusters and the empirical transition frequency between their basins. Figure 3(c) shows the transition frequency (i.e., the number of observed transitions between basins) and Fig. 3(d) shows the transition probabilities in and out of each basin (i.e., the number of observed transitions from each basin into (out of) basin X divided by the total number of transitions into (out of) basin X). Figures 3(c) and 3(d) highlight the asymmetrical nature of the empirical basin transitions. Our results also show an exponential relationship between the size (the number of basin states) and the dwell time of local minima basins [Fig. 3(f)]. Note that the exponential fit in SM Fig. 7 clearly demonstrates that the dwell of the larger basins is exponentially higher than the small and less probable basins [27]. These results demonstrate the significant influence of high-probability local minima with large basins of attraction. To determine if a stochastic process can explain the patterns of state transitions over the estimated energy landscape, we performed a random walk process using the Markov chain Monte Carlo (MCMC) algorithm (see Sec. IV). The results of the MCMC simulations showed that in large-scale clusters, the frequency of transitions between the basins of the local minima closely matched the empirical transition frequencies [Fig. 3(g)]. This relationship was statistically significant, with a linear regression *p* value of 4.6 × 10^−70^ and an *R*^2^ value of 0.91. This indicates that macroscopic state transitions are constrained by the topology of the energy landscape, defined by the relative probabilities of neighboring brain states.

To better understand how the topology of the energy landscape contributes to the observed state transitions, we also examined features of the attractor states and their basins, such as the energy barrier between the local minima and the basin’s size. Specifically, we first constructed the disconnectivity graph that reveals the relationships between the local minima across different energy levels (see SM Fig. 8 [27]). The energy barrier between two local minima is the difference between the local minima and saddle states’ energy (i.e., the lowest energy level connecting two local minima). Our results demonstrate that the energy barriers between minima show a weak relationship with the frequency of transitions between local minima basins, but the linear fit is poor, and the overall correlation is low (see SM Fig. 9).

### E Seizurelike transitions from disorder to order

**Fig. 4:**
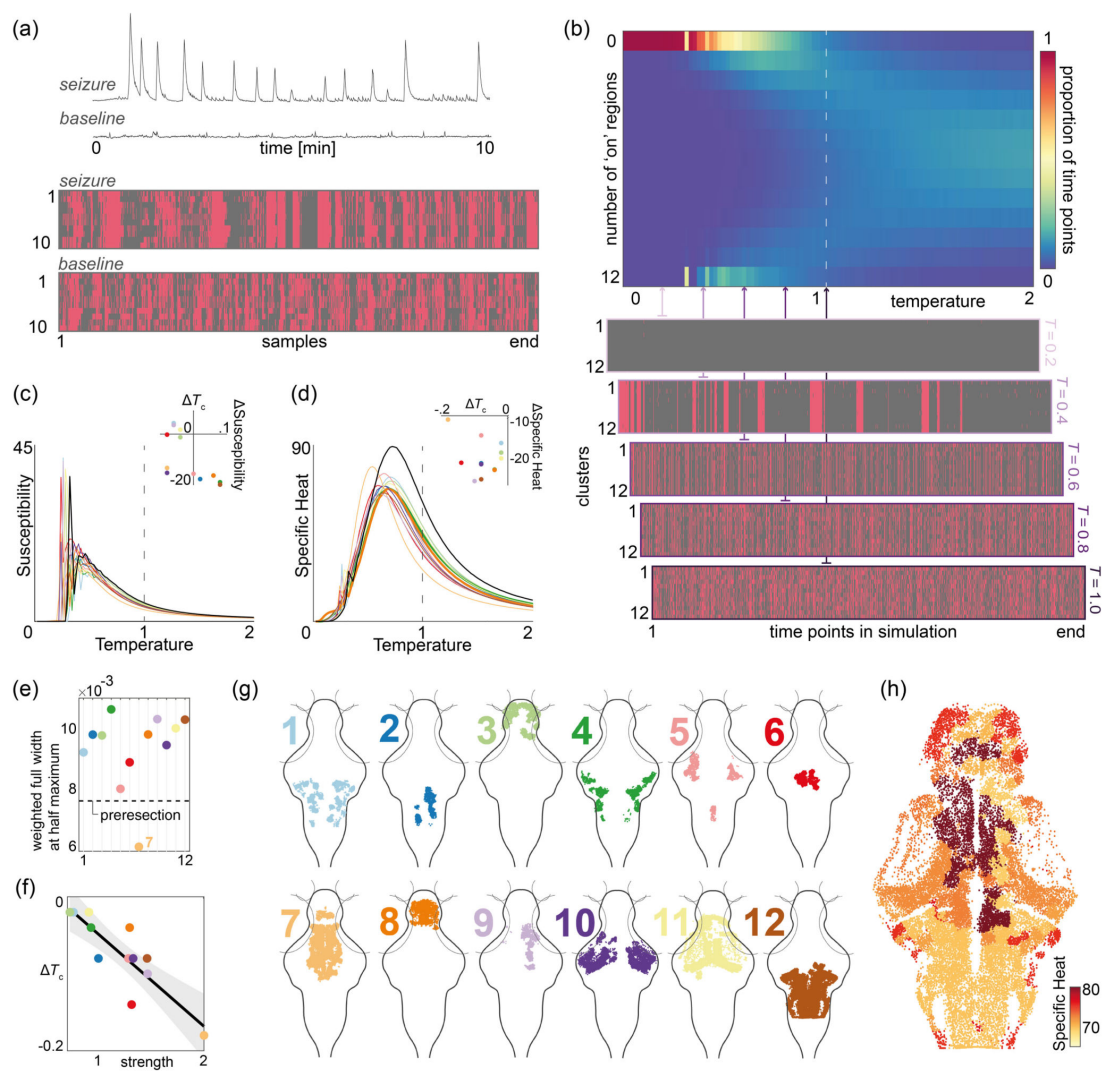
Seizurelike transitions of global activity in the pairwise MEM. (a) Top: example time series of regionally averaged calcium imaging during chemically induced seizures and baseline recordings; bottom: binarized empirically observed state transitions across 10 anatomically defined regions (see SM Fig. 10 for more details [27]). (b) Top: number of regions in the simulated states that are in an “on” or active state, simulated using MCMC algorithm; bottom: the simulated state transitions at five different sample temperatures. Pink indicates clusters in the “on” state, gray indicates clusters in the “off” state. (c),(d) The susceptibility (c) and the specific heat (d) curves before (black) and after (color coded for each cluster) virtual resection. The insets show the change in the peak of the curves following the resections. (e) Full width at half maximum (FWHM) values of the specific heat curves. (f) Postresection change in the critical temperature *T*_*c*_ and the strength of the clusters’ functional connections (i.e., the sum of *J*_*ij*_ rows). (g) Individual functional clusters, projected across all *z* layers onto an *xy* zebrafish brain outline. (h) Postresection values of specific heat maximum mapped onto individual neuron positions, plotted for a single *z* layer.

Next, we utilized the MEM to study transitions between physiological and pathological whole-brain states. For example, epileptic seizures are characterized by paroxysmal transitions of macroscopic brain dynamics into an abnormal dynamical regime [28]. These transitions are a common feature of brain pathophysiology across species [29,30], and represent a huge burden for patients [31]. To study transitions between distinct dynamical states, such as physiological brain activity and hypersynchronous activity seen during epileptic seizures, we took advantage of the characteristic phase transition in the pairwise MEM that occurs between large-scale ordered and disordered states. Such phase transitions are best described by the Ising model, which is mathematically identical to the pairwise MEM. This model was developed in statistical mechanics to explain the transition between ferromagnetic and paramagnetic states in magnetic materials by modeling the interaction of atomic spins. Here, parallel spins have lower energy than antiparallel spins, driving the tendency for atoms to share the same spin, giving rise to large-scale order and ferromagnetism. However, if the temperature in the system is increased above the critical temperature *T*_*c*_, thermal fluctuations disrupt the neighboring spin correlations, causing a disordered mixture of parallel and anti-parallel spins, the paramagnetic phase. This sudden shift from ferromagnetic to paramagnetic behavior with only small changes around *T*_*c*_ is known as a *phase transition*. Changes in the temperature parameter represent a global manipulation of MEM behavior by influencing both *J*_*ij*_ and *h*_*i*_ values, resulting in changes to the dynamics of the system in relation to its phase transition. This has been exploited in a number of previous studies, where changes in the *T* parameter are employed to recapitulate dynamics ranging from single neuron firing in the human brain to social behavior across a group [32–41]. While the *T* parameter does not identifiably map onto specific biological aspects of synaptic function in our model organism, changes in *T* can drive the system into and out of seizurelike states similar to those observed with experimental manipulations through, e.g., drugs that change inhibitory synaptic coupling [Fig. 4(a)]. To validate this assumption, we fitted a MEM to an unrelated dataset of drug-induced seizures in the larval zebrafish brain [16] to estimate the temperature parameter that best captures the shift in brain dynamics from normal function to seizures (see SM Fig. 10 [27]). Inducing this estimated temperature shift as a single perturbation to the system generates dynamics similar to those empirically observed, as demonstrated in more detail in the SM [Figs. 10(b) and 10(c)]. This suggests that the temperature parameter is well suited to capture key changes in brain dynamics during epileptic seizures.

Here, we leverage the same principles to model transitions from physiological to pathological macroscopic brain dynamics and to probe the microscopic interactions from which they arise. Seizure transitions can be induced by globally increasing the excitability of brain networks, for example, through drugs blocking the inhibitory neurotransmitter GABA or enhancing excitatory transmission through glutamate [42,43]. In the more abstract formulation of pairwise MEM, we represent changes in effective synaptic connectivity through changes in the temperature parameter. In the pairwise MEM [Eq. (1)], the temperature is usually set to *T* = 1. However, by dividing the exponent in Eq. (1) by *T* (see Sec. IV), the Ising model can simulate and predict the effects of changing temperature on the large-scale behavior of the system. Here, we modeled a global increase in excitability by a decrease in the temperature of the MEM [Fig. 4(b)]. Mathematically, as the temperature increases, the probability distribution becomes more spread out, allowing for a broader range of energy states to be occupied. This increased spread in energy states corresponds to increased randomness in the system. By performing MCMC simulations under varying values of *T* (binarization threshold *z* = 0; see SM Fig. 11 for *z* = 1 [27]), we demonstrate that reducing the system’s temperature leads to a transition in the global order of the neural activity [Fig. 4(b)]. Namely, the system transitions from disordered activation patterns (i.e., zero mean activation) at higher temperatures (*T >* 1) to highly ordered, hypersynchronous seizurelike states, marked by periods of brainwide activation followed by brainwide inactivity analogous to a postseizure state [44] [e.g., Fig. 4(b), *T* = 0.4]. This demonstrates that the empirically derived MEM may be used to model pathological whole-brain state transitions. It is important to note that the binarization method can significantly change the qualitative behavior of the system. Figure 12(a) in SM shows how the (−1 and 1) state definition does not result in bistability at higher temperatures, and our choice of (0 and 1) in this paper is influenced by this known phenomenon of pair-wise MEM [45]. Next, we aimed to perturb empirically derived MEM parameters to ascertain the contribution of observed biological features in driving physiological-pathological state transitions.

### F Regional contribution to critical dynamics

Since the intrinsic connectivity of brain structures is heterogeneous, each region shapes global dynamics differently. For instance, one could hypothesize that densely connected brain regions with widely distributed connections play an important role in maintaining the system’s dynamics poised between order and disorder, a regime known as criticality. Interestingly, the critical regime may be a favorable regime for brain dynamics [46,47], and brain pathology might emerge as a loss of criticality [48]. Therefore, studying how different brain structures regulate transitions about the critical regime can help us understand how neuronal populations drive optimal and pathological whole-brain regimes. The organizational role of different brain regions can be examined empirically or *in silico* by removing or ablating single regions and their connections, and assessing changes in the macroscopic behavior of the system. Here, we examine how removing individual regions affects the transition between order and disorder in the MEM. This approach allows us to quantify differential distributions of individual brain nodes to whole-brain dynamics. While there are other important, quantifiable network measures that may identify similar nodes as being important [16,49], we focus on the simulation-based virtual resections here specifically as current network-based interventions—such as respective epilepsy surgery—could be conceptualized in a similar framework.

To investigate the transition between order and disorder, we study the specific heat of the system, which measures the change in the average energy due to a slight change in temperature (see Sec. IV). The temperature associated with the peak specific heat [Fig. 4(d)] can be used to identify the critical temperature *T*_*c*_ of a given system. When a region is removed from the model, it no longer influences the rest of the system. This can significantly affect specific heat [Fig. 4(d)], depending on the nature of the interactions with the removed region. For instance, if the interactions with the removed region are strong, removing them effectively reduces global connectivity, which is equivalent to an apparent increase in the temperature of the system, shifting the specific heat curve to the left [Fig. 4(d)]—resulting in critical transitions being achieved at lower temperatures. Figure 4(d) shows changes in the specific heat curves (and, in turn, the critical temperature *T*_*c*_) induced by removing different regions. A decrease in the full width at half maximum (FWHM) of the specific heat curve would correspond to a *sharpening* of the specific heat curves after resection [Fig. 4(e)]. This is seen only when removing cluster 7, suggesting that this intervention makes the transition *sharper*, with a smaller temperature window for critical behavior. Cluster 7 corresponds to the diencephalon, which contains the thalamus and hypothalamus, among other regions, with known roles for homeostatic regulation and sensorimotor integration. This finding indicates that the connectivity of the diencephalon supports a broad range of network states that reside in the vicinity of the critical regime. The above-mentioned choice of removing the *J*_*ij*_ elements to model the postrespective behavior of the system is motivated by our results that *J*_*ij*_ captures the underlying structural connectivity, and we aimed to avoid for regional resection to affect other pairwise structural relationships. As seen in SM Fig. 13, an alternative approach of ignoring a single region (for example, the highly connected cluster 7) and refitting the model results in an artificial increase in a large number of *J*_*ij*_ elements [27].

Interestingly, our results show that the specific heat decreases and peaks at lower temperatures for all resections compared with the full system. Therefore, the connections provided by each region tend to push the system closer to criticality compared to a system lacking this region, but with all other parameters being equal (e.g., the same connection weights between all other regions). However, if the interactions with the removed region are weak (e.g., clusters 1 and 3), removing them might only have a negligible effect on the system, resulting in a minor change in the specific heat. In fact, we observed a close relationship between the overall strength of a cluster’s functional interactions and its postresection effect on the critical temperature [Fig. 4(f)]. In SM Fig. 12, we provided the resection results for the (−1 and 1) state definition [27]. Note that although there are some differences in the Δ *T*_*c*_ and Δ specific heat, overall, the results are qualitatively similar and highlight the same regions having the most impact following the resection.

Removing a region from the model can also affect other system properties, such as magnetization and susceptibility. The susceptibility measures the change in the net magnetization (i.e., average activation across all regions) due to small changes in the external field *h*_*i*_, which roughly corresponds to regional excitability or activation propensity (see Sec. IV). Susceptibility quantifies how much network states change in response to external perturbations, which in a brain may be related not only to the dynamic properties but also to information transfer [50]. Therefore, network alterations that significantly impact the susceptibility of a brain network at a given temperature may significantly alter the information processing properties. The postresection susceptibility measures echo the specific heat results [Fig. 4(c)] and identify similar region-specific effects. Finally, we also explored the effect of increasing the cluster activation binarization threshold on the above-mentioned results. Figure 14 of SM shows that the resection of some of the similar clusters, such as cluster 7, also results in the biggest change in the susceptibility and specific heat curves at higher binarization thresholds (e.g., *z* = 1) [27]. Susceptibility and specific heat capture key relationships between macroscopic network dynamics and stochasticity in the system parametrized by the temperature. Here, we use these measures to summarize the differences in dynamic behaviors induced by modifications of the network structure through the simulated virtual resections. Some of these quantifiable features may be related to functional properties of the resultant brain networks [50,51], which in the future may be used to establish testable hypotheses regarding brain dysfunction from simulations based on empirically derived MEMs as presented here. Taken together, these findings suggest regional specificity in regulating whole-brain critical dynamics, with hublike regions exhibiting particularly strong control over the critical regime.

## III Discussion

### A Capturing whole-brain dynamics in the larval zebrafish brain through minimal models of pairwise interactions

Neuronal systems display complex population dynamics that unfold as observable patterns of whole-brain activity. Whether these patterns emerge from simple pairwise interactions between brain regions remains a central open question. The pairwise MEM approach presented here accounts for the observed covariance (i.e., functional connectivity) between regions but remains explicitly agnostic to all higher-order interactions. The MEM includes two key terms: intrinsic neuronal activity (*h*_*i*_) and pairwise interactions (*J*_*ij*_). Here, we show that the probabilities of collective brain states can be described by pairwise MEM, indicating that macroscopic features of neural activity can arise from fine-scale interactions.

Tested in an empirical dataset with single-neuron resolution, previous work in zebrafish had already demonstrated that simple pairwise interactions between neuronal ensembles could reproduce statistical characteristics of whole-brain dynamics [20]. In the dataset presented here, we aimed to predict the probabilities of specific whole-brain states at coarse-grained scales. In finer-scale, higher-dimensional data, the pairwise MEM fails to fit the neuronal data accurately in our experiment and previous reports [10,52]. This suggests that while microscopic dynamics of individual neurons are difficult to predict with sufficient accuracy at the whole-brain scale, their coarse-grained activity can be characterized by the interactions between neuronal populations or brain areas.

Including higher-order correlation to MEM models of brain dynamics can yield overall improved model fits [53,54], compared to the model presented here, and are less easy to interpret against complementary measures of pairwise neuronal connectivity. Some of the higher-order interactions may represent specific synaptic and extra-synaptic coupling mechanisms that are not well described by pairwise interactions or capture nonlinear features of regional interactions that are otherwise not well described by the pairwise MEM model. However, with increasing model parameters, the models may perform better on a given dataset but may reduce in generaliability—i.e., they may be overfitting. Here, we chose to focus on pairwise interactions to test generalizability across datasets in evaluating the relationship between the *J*_*ij*_ matrix and structural connectivity. Such a mapping between structural connectivity and functional recordings becomes more challenging at higher orders of interactions.

In addition to modeling the probabilities of individual states, we also wanted to test whether the MEM can further characterize dynamics such as the transition between brain states. For this, we simulated brain activity sequences as a random walk on the energy landscape between neighboring brain states, as defined by the pairwise MEM. This simulation predicts the dwell time in individual attractor basins, as well as the frequency and probability of transitions between basins, with high accuracy. This finding suggests that resting state macroscopic brain state transitions are a natural consequence of the energy landscape over the states induced by the maximum entropy principle.

The optimum scale of coarse graining that allows for both accurate predictions of brain state probabilities and for the models to map onto known structural connectivity patterns likely depends on the exact nature of the data. MEM approaches have demonstrated excellent fits to dynamics ranging from spiking behavior in neuronal populations [55] to whole-brain functional MRI patterns in the human brain [24]. The applicability of MEM approaches across such diverse scales suggests that pair-wise interactions underlie dynamics at multiple scales and that the optimum scale for a given dataset can be identified empirically through clustering or coarse graining.

While zebrafish larval imaging allows for unprecedented insight into whole-brain dynamics, these are not fully resolved at the microscale. Specifically, there is no one-to-one mapping between neuronal firing rates and calcium traces [56,57], and limitations of the methods may impair recordings from certain brain regions or cell populations. At the coarse-graining scale represented in our analyses, these issues are less likely to significantly impact findings, but in analyses aimed at single-cell resolution, these may limit biological interpretation of the findings at the scale of individual neuronal firing.

There is a tension here in our understanding of brain dynamics: Simple models can comprehensively describe large-scale brain activity patterns, yet neuronal assemblies encode temporally and spatially precise information in ways that can be modulated through the activity of only a few isolated neurons [58]. Identifying how large-scale patterns of whole-brain dynamics, as described here, shape information processing and neuronal responses at the microscale is a current research area of much interest, and simple organisms whose neuronal activity can be mapped across scales play an important role in further exploring this relationship [59,60].

### B Macroscale pairwise interactions reveal underlying structural connectivity

Neuronal activity and pairwise synaptic connectivity can be measured in the larval zebrafish brain at single-cell resolution, allowing unprecedented access to functional and structural connectivity at brainwide scales [23,61]. The analysis presented here adds to existing evidence indicating a strong correlation between structural measures of the strength of physical synaptic interactions and functional (i.e., model-derived) estimates of effective interactions. This structure-function coupling has been reported across diverse neuronal data recording modalities [24,25,62], including calcium imaging in the larval zebrafish [20]. In our analysis, pairwise interactions between clusters of neurons quantified in the *J*_*ij*_ matrix closely resemble the strength of pairwise interactions between regions as measured by axon count (i.e., the structural connectivity). This effect is robust when modeling neuronal clusters spanning orders of magnitude in size.

However, the model-based estimates of pairwise interactions do not capture underlying structural connectivity equally well across all parameter choices. Indeed, there appears to be a characteristic scale, in terms of the number of neural clusters, at which the effective MEM interactions *J*_*ij*_ performed best at identifying above-threshold structural connections between brain regions: At the relatively coarse scale of 12 clusters consisting of an average of 1623(±392) to 17740(±3122) neurons each, the majority of large between-region structural connections could be identified from the MEM interactions *J*_*ij*_. There is, therefore, an apparently closer structure-function coupling at coarser scales of the analysis than at the neuronal level. This might indicate that averaging over some microscale heterogeneity at the neuronal level results in a mean-field approximation of the structural connectivity, which constrains the coarse-grained activity patterns accordingly.

Biologically, the majority of neuronal interactions are mediated through direct neuron-to-neuron directed signaling at the synapse [63]. However, other types of coupling—including ephaptic transmission, extracellular fields, metabolic regulation [64,65], and more complex patterns of synaptic connections between three or more neurons—may deviate from a simple pairwise model. Many of the neuronal interactions that are not mediated through typical pairwise synaptic coupling are highly local, affecting mostly neighboring neurons rather than mediating fast, between-region interactions [66]. Thus, coarse graining single-cell data to functional clusters may separate pairwise synaptic coupling that governs between-region connectivity from higher-order interactions that are subsumed in the within-cluster average. Furthermore, while single-neuron dynamics are highly nonlinear [67], coarse-grained brain dynamics are well approximated by linear models [68] suggesting that coarse graining may improve model performance for models based on linearity assumptions.

### C States of global hypersynchrony can emerge without coupling changes

Complex biological systems like the brain characteristically show emergent patterns of behavior that are shaped by the function and dysfunction of its constituent parts. Abstract physical models like the one presented here have been useful in defining minimal sets of constraints under which neuronal population activity patterns may emerge in the zebrafish brain under specific conditions [69,70]. Such emergent patterns can arise during physiological brain function, e.g., during the transition between wakefulness and slow wave sleep [71,72], or during epileptic seizures. Epileptic seizures are characterized by hypersynchronous neuronal firing that arises from a wide range of possible neuronal or synaptic dysfunction [73]. Seizurelike patterns have been demonstrated to emerge even in minimal Ising models under certain conditions [74,75], and there is a growing interest in how phenomenological descriptions of seizure patterns may aid the understanding of which pathophysiological mechanisms may be crucial in their etiology [29]. Rapid physiological and pathological transitions between asynchronous and hypersynchronous brain states occur without significant structural changes in synaptic coupling and from apparently gradual transitions in physiological parameters. We, therefore, aimed to test with our modeling approach whether hypersynchronous patterns can arise from gradual modulations of existing between-region coupling parameters.

Using the MEM model, we can show that modulation of intrinsic excitability (modeled here as a temperature parameter *T*), without changes in between-node coupling (i.e., without changes in the *J*_*ij*_ matrix), can lead to a sudden transition between asynchronous and hypersynchronous states. Specifically, increasing the temperature in the model flattens the probability distribution, effectively reducing the influence of pairwise structure that favors low energy states and increasing the randomness in the activity patterns. These findings build on similar modeling of sleep-wake transitions in human brain data [76]. Biological systems may demonstrate similar transitions through diffusely distributed modulatory neurotransmission (e.g., through neuromodulators such as acetylcholine and noradrenaline that play a significant role in sleep-wake transitions).

Notably, the simulated activity patterns in the MEM-derived model of zebrafish brain’s spontaneous activity do not show a gradual transition from asynchronous to hyper-synchronous activity but rather show a sudden transition with the gradual change in the *T* parameter resulting in significant changes of whole-brain activity patterns around the transition region. In systems poised at the boundary between these regimes, one would expect the system behavior to be very sensitive to even small changes in the *T* parameter. In fact, these findings provide evidence of state transition dynamics during drug-induced seizures in larval zebrafish, which demonstrate that over short timescales, the dynamics can transition from critical to a chaotic, supercritical regime [22]. Epileptic seizures are classically considered to represent hypersynchronous activity with population-level neurophysiological signatures suggestive of excessively ordered activity that is synchronized across multiple brain regions. However, with the advent of large-scale calcium imaging, this view has been challenged, with many findings suggesting highly variable or even chaotic patterns at the scale of single neurons to occur during epileptic seizures [22,77,78]. Future work will have to address the appropriate scale at which abnormal brain dynamics are best captured in epileptic seizures in order to allow for the prediction of the efficacy of different scale interventions.

### D Alterations affect the response to perturbation

Studying phase transitions and critical phenomena in regular lattices has led to key insights in statistical physics [79,80]. However, biological networks like the brain have connectivity patterns that are unlike lattices. These complex networks exhibit nonuniform connectivity patterns, such as power-law degree distributions in scale-free networks [81]. As a result, phase transitions and critical phenomena in complex networks are significantly different from those in regular lattices. The Ising model, in particular, exhibits anomalous phase transitions in complex scale-free [79,82–85], modular [86–88], and core-periphery networks [89].

For instance, unlike the first-order phase transitions of lattice structures, random scale-free networks display a second-order phase transition. In addition, the critical temperature for the onset of ferromagnetic ordering depends on the network’s degree distribution, which determines the universality class of the phase transition [79]. Moreover, modular structures in complex networks can give rise to metastable phases in both the equilibrium and nonequilibrium regimes, marked by the coherent alignment of intracommunity spins and misaligned intercommunity spins [86–88].

Consequently, modifying brain networks can have non-trivial effects on overall dynamics. For example, removing a single region from the zebrafish brain network can drastically affect global dynamics. There is some previous evidence that inferences on global network dynamic out-comes can be drawn on *virtual* resection simulations. For instance, densely connected regions tend to have an outsized influence and their removal results in significant changes in large-scale dynamics of the system as a whole [90–92]. Moreover, resection analyses are starting to play a role in cognitive models, allowing hypothesis generation for processes such as language, attention, and memory [93]. Our methodological choice of removing *J*_*ij*_ elements to model a postresection system is motivated by our observation that *J*_*ij*_ captures the underlying structural connectivity between regions, and removing a single region should not change the structural connectivity, and consequently, *J*_*ij*_ elements between remaining regions. Nevertheless, future empirical work should verify our assumptions by inferring and comparing the model parameters preablation and postablation of a brain structure.

Similarly, resection analysis can also be used to study the robustness and vulnerability of networks to perturbations [94]. Here, we examined the transition between dynamic regimes of the system toward globally hypersynchronous states as a proxy for evaluating the resilience of an altered system to pathological dynamics as seen, for example, during epileptic seizures. Specifically, evaluating post *virtual resection* networks for changes in sensitivity to perturbation through specific heat and susceptibility measures reveals node-specific changes to whole-network sensitivity to small perturbations following the removal of single network nodes. In our model and previous findings these node-specific effects seem to be mediated through the nodes’ particular connectivity patterns [95,96]. It is critical to note that although the major functional hubs are similarly identified in the alternative (−1 1) state definition, the choice of (0,1) in our work is based on the superficial similarity of the qualitative bistable behavior of the system at higher temperatures. Therefore, future empirical work should verify our methodological choice and demonstrate which state definitions result in more accurate predictions of the postrespective system changes.

These findings highlight the importance of functional hub brain regions in maintaining the overall function and resilience of the zebrafish brain network. The approach taken here allows us to demonstrate that the virtual resection of functional clusters involving the optic tectum has the largest impact on overall resultant network dynamics, mirroring its essential role in guiding zebrafish larval behavior. Previous studies have identified overlapping regions as central hubs maintaining resting state dynamics, with rich, effective connectivity to most other brain regions [16]. Similar regions—that is, nodes involving the midbrain and tectum—have already been implicated in epileptic seizure generation through recordings of seizure activity in zebrafish [16,49]. In future work, we are hoping to verify our theoretical findings here with targeted experiments altering network topology in the larval zebrafish brain. Our framework now provides a modeling approach to infer network structure and behavior from dynamic observations of complex systems. Similar approaches in the future may inform the development of targeted interventions in complex networks, such as surgery or regional neuromodulations for neurological and psychiatric disorders, such as epilepsy [90,97,98], or disorders of consciousness [40,99,100]. In addition, our study demonstrates the usefulness of resection analysis in studying the functional connectivity of complex networks, capable of providing valuable and empirically verifiable predictions. These results offer a phenomenological description of large-scale epileptic seizures in a hugely simplified model in which neurons have binary states and limited interaction. The pathophysiology of epileptic seizures, even in simple model organisms like the zebrafish, is likely to involve more complex alterations of normal brain function that are not captured in the level of abstraction provided by the current model. However, the simplified model presented already recapitulates key aspects of seizurelike dynamics and allows us to interrogate contributions of individual brain regions and, through the virtual resection approach, generate hypotheses for future empirical testing.

## IV Materials and Methods

### A Datasets

In order to capture whole-brain activity at cellular resolution in a vertebrate brain, we used resting state whole-brain calcium imaging datasets recorded in larval zebrafish at day six postfertilization using light sheet imaging. Dataset acquisition is explained in detail in Ref. [101] and was accessed through the public repository [102]. Briefly, images were recorded using a light sheet microscope at two volumes/second in transgenic larval zebrafish expressing the genetically encoded calcium indicator *GCAMP6f* pan-neuronally within the cell nucleus. This approach allowed automatic cell detection [103], resulting in approximately 80 000 individual neuron calcium fluorescence traces per fish. For the analysis, we used resting state segments, recording spontaneous activity under homogeneous background illumination conditions without changes in visual stimuli for approximately 5–8 minutes recorded for 11 individual larvae. Individual zebrafish brains were registered to an atlas template [104], and standardized atlas-based locations are used for subsequent analyses.

### B Pairwise maximum entropy model

To bridge the gap between microscopic details and macroscopic predictions about the likelihood of different neural states, we employ a pairwise maximum entropy model. By maximizing the entropy of the distribution over activity states, we arrive at a prediction that is optimally unbiased, given a set of microscopic details about the system. Here, we constrain the average activities and pairwise correlations between different brain regions, thus yielding the pairwise MEM. To fit the pairwise MEM, we calibrate the external fields *h*_*i*_ and functional interactions *J*_*ij*_ such that the model matches the activation and coactivation rates observed in the real system. From these microscopic constraints, the pairwise MEM makes large-scale predictions about the large-scale zebrafish brain dynamics.

We applied dimensionality reduction to all fish datasets using *k*-means clustering based on cell position. This process resulted in the identification of 1000 clusters of neurons, determined by their spatial proximity. Subsequently, these spatial clusters were grouped into a smaller number of functional clusters (ranging from 8–16, 32, 64, and 128) by analyzing them based on their covariance. The covariance between clusters was calculated from the average calcium traces of all neurons within each cluster.

We analyzed 83 minutes of recordings from 11 zebra-fish larvae. At each time point *t*, the activation state of the system is defined by the binary vector $\sigma^{t}=\left[\sigma_{1}^{t},\sigma_{2}^{t},\ldots ,\sigma_{N}^{t}\right]$, where $\sigma_{i}^{t}$ is the binarized average calcium trace of cluster *i* at time *t* and *N* is the total number of clusters. Specifically,$\sigma_{i}^{t}=1(-1)$ for activation above (below) the *z*-scored average calcium time series at various thresholds (*Z* = 0, 0.5, 1, 1.5, 2). Calcium fluorescence is a continuous measure of neuronal activity, which is indirectly linked to neuronal firing rate and, at the population level, is highly correlated with local field potentials [105]. To model whole brain state transitions with data that are feasible to record empirically, there is a trade-off: Recording length limits the amount of time the brain is captured, particularly in rare states, resulting in noisier estimates of state transition probabilities for high-dimensional state spaces. Based on previous work [24], we elected to reduce the dimensionality of the data and resultant states and binarize the time series as described below. To obtain the empirical activation rate of cluster *i*, we calculated the average of $\sigma_{i}^{t}$ over all time slices. This is represented as $\sigma_{i}=(1/T)\Sigma_{t=1}^{\tau }\sigma_{i}^{t}$, where *τ* is the number of time slices. Similarly, the empirical correlation between cluster *i* and *j*, denoted as ⟨*σ*_*i*_*σ*_*j*_⟩, is defined as the average of the product of $\sigma_{i}^{t}$ and $\sigma_{j}^{t}$ over all time slices, which is calculated as $(1/\tau )\Sigma_{t=1}^{\tau }\sigma_{i}^{t}\sigma_{j}^{t}$.

The pairwise MEM is constrained such that the model averages ⟨*σ*_*i*_⟩_*m*_ and ⟨*σ*_*i*_*σ*_*j*_⟩_*m*_ match the empirical values of ⟨*σ*_*i*_⟩ and ⟨*σ*_*i*_*σ*_*j*_⟩. The probability distribution over states that satisfies these constraints and maximizes the entropy is the Boltzmann distribution [7]:

(2) $$P(\sigma )=\frac{1}{Z}e^{-E(\sigma )},$$

where *Z* is the normalizing partition function, given by

(3) $$Z=\underset{\sigma }{\sum }e^{-E(\sigma )},$$

and *E*(*σ*) is the energy of this state, given by

(4) $$E(\sigma )=-\sum_{i=1}^{N}h_{i}\sigma_{i}-\frac{1}{2}\sum_{i,j=1}^{N}J_{ij}\sigma_{i}\sigma_{j}.$$

The parameters *h*_*i*_ represent the bias toward activation and *J*_*ij*_ represent the functional interaction between clusters *i* and *j*. To fit the pairwise MEM, one adjusts the parameters *h*_*i*_ and *J*_*ij*_ using a gradient descent algorithm [106] until the empirical averages ⟨*σ*_*i*_⟩ and ⟨*σ*_*i*_*σ*_*j*_⟩ match the model averages.

The aforementioned methods are computationally expensive when the dimensionality is higher than *N* = 15. To overcome this hurdle, for higher dimensions, we used a pseudo-likelihood maximization algorithm instead of the likelihood maximization approach. The MATLAB scripts provided by Ref. [107] were used to estimate the model parameters for dimensions larger than *N* = 15. In the pseudo-likelihood maximization scheme, the goal is to solve the following equation:

(5) $$(h,J)=\underset{h,J}{\text{argmax}}ℒ(h,J),$$

where the pseudo-likelihood function ℒ(*h, J*) is defined as

(6) $$ℒ(h,J)\approx \prod_{t=1}^{t_{\text{max}}}\prod_{i=1}^{N}\tilde{P}\left(\sigma_{i}(t)|h,J,\sigma_{/i}(t)\right),$$

where $\tilde{P}$ represents the Boltzmann distribution for a single spin (i.e., cluster) *σ*_*i*_ given that the other *σ*_*j*_ (*j* ≠ *i*) values are fixed to *σ*_*/i*_(*t*) ≡ [*σ*_1_(*t*), …; *σ*_*i*−1_(*t*); *σ*_*i*)1_(*t*), …; *σ*_*N*_(*t*)]. Therefore, $\tilde{P}$ is given by

(7) $$\tilde{P}\left(\sigma_{i}(t)|h,J,\sigma_{/i}(t)\right))=\frac{e^{\left(h_{i}\sigma_{i}+\Sigma_{j=1}^{N}J_{ij}\sigma_{i}\sigma_{j}(t)\right))}}{\Sigma_{\sigma_{i}^{\prime }=1,0}e^{\left(h_{i}\sigma_{i}^{\prime }+\Sigma_{j=1}^{N}J_{ij}\sigma_{i}^{\prime }\sigma_{j}(t)\right))}},$$

The above method uses a mean-field approximation that disregards the influence of one variable on another. However, the estimator obtained by maximizing the pseudo-likelihood converges to the maximum-likelihood estimator as the number of time steps *t*_max_ increases, as per Ref. [108]. To estimate the model’s parameters, *h* and *J*, we use a gradient descent scheme, which updates the parameters by comparing the empirical mean and correlation values to the mean and correlation values predicted by the model. The update equations are as follows:

(8) $$h_{i}^{\text{new}\;}-h_{i}^{\text{old}\;}=\epsilon \left({\langle \sigma_{i}\rangle }_{\text{empirical}}-{\langle \sigma_{i}\rangle }_{\tilde{P}}\right)$$

and

(9) $$J_{ij}^{\text{new}\;}-J_{ij}^{\text{old}\;}=\epsilon \left({\langle \sigma_{i}\sigma_{j}\rangle }_{\text{empirical}\;}-{\langle \sigma_{i}\sigma_{j}\rangle }_{\tilde{P}}\right),$$

where the superscripts new and old represent the parameters after and before a single updating step, respectively, *ϵ >* 0 is the learning rate, and ${\langle \sigma_{i}\rangle }_{\tilde{P}}$ and ${\langle \sigma_{i}\sigma_{j}\rangle }_{\tilde{P}}$ are the mean and correlation with respect to distribution $\tilde{P}$ [Eq. (5)] and are given by

(10) $${\langle \sigma_{i}\rangle }_{\tilde{P}}=\frac{1}{t_{\max}}\sum_{t=1}^{t_{\text{max}}}\text{tanh}\left[h_{i}+\sum_{j^{\prime }=1}^{N}J_{ij^{\prime }}\sigma_{j^{\prime }}(t)\right]$$

and

(11) $${\langle \sigma_{i}\sigma_{j}\rangle }_{\tilde{P}}=\frac{1}{t_{\text{max}}}\sum_{t=1}^{t_{\text{max}}}\sigma_{j}(t)\text{tanh}\left[h_{i}+\sum_{j^{\prime }=1}^{N}J_{ij^{\prime }}\sigma_{j^{\prime }}(t)\right],$$

respectively. For more details regarding the likelihood and pseudo-likelihood maximization algorithms and scripts, see Ref. [107].

### C Dimensionality reduction and structure-function coupling

The correlation between structural and functional connectivity is a defining characteristic of brain networks in humans [93,109] and other species (e.g., mice [110], rats [111], and monkeys [112]). Recent studies have provided further evidence of structural and functional similarity in the zebrafish brain [20]. In light of these observations, we aimed to reduce the dimensionality of the functional dataset by selecting a resolution of functional parcellations that maximizes the similarity between the estimated *J*_*ij*_ and the structural connectivity matrix (total tract count between large-scale clusters). Our findings reveal the high similarity between structural and functional matrices across 8–16, 32, 64, and 128 scales. However, the highest similarity, as measured by the correlation between the two matrix modalities and the AUC for the detection of binarized structural connections between regions from *J*_*ij*_ weights, was consistently identified at *N* = 12 regions and functional binarization threshold of 1% and 30% of the maximum weight of the structural connectivity matrix (see SM Fig. 4 [27]).

### D Evaluating the accuracy of the pairwise MEM

We used information-theoretic approaches to evaluate the fit of our model. A pairwise maximum entropy model better fits observed dynamics than a first-order (independent) model since it considers not only regional activations but also pairwise correlations between regional time series. These additional considerations lead to a lower uncertainty or entropy in the second-order model than in the first-order model. Intuitively, increasing the order of the model will monotonically decrease the entropy closer to the true entropy, which can be measured empirically. We can measure this entropy difference using the multi-information, *I*_*N*_ = *S*_1_ − *S*_*N*_, given by the difference between the first-order model entropy *S*_1_ and the empirical entropy of the data *S*_*N*_. In the context of our study, the multi-information measures the total amount of correlation in brain signals independent of higher-order correlations. To evaluate the performance of the pairwise MEM, we asked whether the reduction in entropy following the incorporation of pairwise interactions in the model, *I*_2_ = *S*_1_ − *S*_2_, captured the majority of the total multi-information. In other words, we quantified the performance of the pairwise MEM as the fraction of the multi-information captured by the second-order model, *r* = *I*_2_/*I*_*N*_, where *r* can range between 0 and 1. Our results showed that pairwise interactions accounted for a large portion of the multi-information, approximately 84%.

### E Identifying local minima and basins of the energy landscape

We defined the energy landscape by the network of cluster activation states and their energy values. In this landscape, the neighboring states are only one hamming distance apart, meaning that adjacent states are identical up to the activity of one brain region [113]. To identify the local minima or attractor states, using a steep search algorithm, we exhaustively searched the entire landscape to find the states with energies lower than all their adjacent states. Next, to find the states that belong to the basin of each local minima, we first start at a given state *σ* and iteratively move to a neighboring state *σ*′ if *E*(*σ*′) *< E*(*σ*). We continue tracing this path until we reach a local minimum where no neighboring states have lower energy (similar to Refs. [24,106]). We consider the basin size of this local minimum to be the ratio between the number of basin states to the total number of possible states.

We were particularly interested in identifying the obstacles that impede the transition between the different basins of attraction. To investigate these transitions, we employed a method that involved removing the highest energy state from the energy landscape, along with the edges linking it to its neighboring states. We then determined whether each pair of local minima was still connected by a path in the reduced landscape. We repeated this process until we discovered the state whose removal causes one or more local minima to be disconnected within the landscape (i.e., the saddle states). We repeated this process until we arrived at a reduced landscape where all the local minima were disconnected, and we could identify all the saddle states. The disconnectivity graph in SM Fig. 8 was created using the identified energy values of the identified local minima and saddle states [27].

We calculated the *asymmetric* energy barrier [114] between each pair of local minima by taking the difference between the energy of the saddle state and the energies of the two minima. We then defined the *symmetric* energy barrier between two local minima as the minimum between the two asymmetric energy barriers. If the energy barrier between two local minima is high, then we hypothesize that the rate of transition between them is low, at least in one direction [24]. However, our results did not show a strong relationship between estimated energy barriers (symmetric and asymmetric) between local minima states and the empirical basin transition probabilities (see SM Fig. 9 [27]). The energy barrier is calculated based on two states: the local minima and the saddle state. However, we used this measure as a proxy for the transition probability between states belonging to different basins. We believe this approximation fails to capture other important factors, such as the shape of the basin (e.g., how steep the gradient is toward the local minima) or the smoothness or roughness of the basins. Consequently, the energy barrier does not accurately capture the basin transition probabilities.

### F Simulating state transitions

To better understand the spontaneous patterns of activations and state transitions, we simulated the large-scale dynamics as a random walk process over the estimated energy landscape using a Markov chain Monte Carlo with Metropolis-Hastings algorithm [114–116]. In this method, the activation state *σ* is allowed an isometric transition to one of *N* neighboring states. Next, the actual transition from *σ* to *σ*′ occurs with probability *P*(*σ*′j*σ*) = min[1; *e*^*E*(*σ*)−*E*(*σ*′^)]. We conducted a 10^6^ step (plus 3^4^ initial steps) walk with randomly chosen initial states. Next, we removed the initial steps to thermalize and ensure the independence of results from the initial conditions. Finally, we construct a trajectory between different basins of attraction from state walks. By comparing the basin transition probabilities in simulations and experiments, we can assess the extent to which the dynamics of the proposed random walk model align with those observed in the brain.

### G Evaluating the thermal properties of the system

Distinct dynamical states have been observed across multiple spatial scales in neurophysiological recordings and are of interest, particularly in the context of pathological dynamics such as those observed in epileptic seizures. The Ising model is a well-established model capturing many features observed during epileptic seizures [117] and is mathematically equivalent to the MEM estimated in this paper. Since the MEM, as illustrated here, is a generative model, simulations can explore model behavior following a range of perturbations. Here, we will use a number of perturbations that, in some ways, resemble pathophysiological features seen in pathological brain states to demonstrate how this modeling approach may help identify empirically testable hypotheses based on observed network behavior.

Pathological processes affecting whole-brain dynamics—such as those relating to the emergence of epileptic seizures—often have multiple interacting effects on neuronal dynamics. During seizures, there are well-described changes in mostly local within-region inhibitory coupling [118], as well as excitatory between-region coupling [119]; Additionally, neuronal function during seizures is altered through neuromodulatory coupling and local changes in the homeostatic environment [120,121]. No single model parameter equates to the sum of these effects. However, based on previous literature [70], we chose to model the collection of physiological changes through changes in the single temperature parameter *T*. This scales the dependence on structural connectivity versus intrinsic excitability for each region, akin to neuromodulatory mechanisms rescaling the net effective connectivity between regions. At the same time, *T* affects *h*_*i*_, capturing changes in intrinsic excitability, which may represent alterations in intrinsic, within-region inhibitory-excitatory connection balance.

The thermal behavior of the MEM was simulated using an MCMC algorithm. We account for the temperature of the system using the following variant of the Boltzmann distribution over activity states (4):

(12) $$P(\sigma )=\frac{1}{Z}e^{-(1/T)E(\sigma )},$$

where *T* is the temperature and the partition function is now given by

(13) $$Z=\underset{\sigma }{\sum }e^{-(1/T)E(\sigma )}.$$

We initialized the simulation with a random initial state followed by 1 030 000 random walks. Similar to the above-mentioned state transition simulations, The first step of the simulation involved discarding the initial 30 000 steps as thermalization steps and then down sampling the remaining walks every 500 samples. The system’s final state at each temperature step was then used as the initial state for the next lower temperature step. The simulations were repeated at different temperatures, starting at the highest temperature of *T* = 2 and gradually reducing the temperature to *T* = 0.

We used the fluctuation-dissipation theorem [122] to calculate the specific heat and susceptibility. This theorem relates the fluctuations of the energy to the changes in temperature, such that the specific heat is given by

(14) $$C=\frac{\langle E^{2}\rangle -{\langle E\rangle }^{2}}{T^{2}},$$

where *E* is the energy, *T* is the temperature, and angle brackets indicate an average in the MEM. Similarly, the susceptibility is given by

(15) $$\chi =\frac{\langle M^{2}\rangle -{\langle M\rangle }^{2}}{T},$$

where *M* =Σ_*i*_ *σ*_*i*_ is the magnetization of the system. In infinite systems, these models undergo well-characterized phase transitions, which are shown to be preserved in larger-scale finite systems [123]. In the smaller-scale system under consideration here with the number of nodes *N* = 12, we will refer to transitions between dynamical regimes rather than phase transitions.

Pathological dynamics, as is observed, for example, during epileptic seizures, are associated with abnormal patterns of whole-brain activation. Calcium imaging in larval zebrafish models of epileptic seizures has been shown to be characterized by sustained, spatially wide-spread excessive neuronal activity [22]. At the level of individual neurons, these abnormal dynamics can be modeled as deviations from a critical regime. Based on these observations, we sought to identify the conditions under which the coarse-grained model investigated here demonstrates similar transitions between dynamic regimes. To understand the role of different regions in the system’s thermal behavior, for each region, we removed all of the connections to that region. We then compared the specific heat and susceptibility curves obtained from the resection simulation to the original system to understand how the resected region changes the critical behavior of the system. Specifically, we measured the shift in the peak of the specific heat and susceptibility curves.

## Supplementary Material

Supplementary Materials
