# Supplementary material for: Spontaneous Brain Activity Emerges from Pairwise Interactions in the Larval Zebrafish Brain
Source: Phys Rev X. Author manuscript; Available in PMC 2025 Feb 9. (PMC7617382; doi:10.1103/PhysRevX.14.031050)
Supplement: Supplementary Materials [file EMS203091-supplement-Supplementary_Materials.pdf]

Supplementary document for "Spontaneous brain activity emerges from pairwise interactions in the larval zebrafish brain"

Richard E. Rosch
Department of Clinical Neurophysiology, King's College Hospital NHS Foundation Trust, London, United Kingdom

Dominic R. W. Burrows
MRC Centre for Neurodevelopmental Disorders, King's College London, London, United Kingdom

Christopher W. Lynn
Initiative for the Theoretical Sciences, Graduate Center,
City University of New York, New York, USA and
Joseph Henry Laboratories of Physics and Lewis–Sigler Institute for Integrative Genomics, Princeton University, Princeton, NJ, USA

Arian Ashourvan
Department of Psychology, University of Kansas, Lawrence, United States of America[*]
(Dated: March 2023)

CONTENTS

SI1. Evaluation of k-means clustering across different cluster sizes. 1
Calinski-Harabasz criterion 1
Davies-Bouldin criterion 2
Silhouette Analysis 2

Supplementary Information (SI) figures 2

References 2

[*] Correspondence to: ashourvan@ku.edu

SI1. EVALUATION OF K-MEANS CLUSTERING ACROSS DIFFERENT CLUSTER SIZES.

We used several criteria for evaluating the quality of k-means functional clusters. These criteria help to assess how well the data points are separated into distinct clusters and how meaningful these clusters are. Therefore, these clustering evaluation criteria can help determine the optimal number of clusters and assess the quality of the clustering results.

Calinski-Harabasz criterion

We utilized the Calinski-Harabasz criterion [1], which is also known as the variance ratio criterion, to determine the optimal number of clusters in our data. This criterion is calculated using the following formula:

$$VRC = \frac{SS_B}{SS_W} \times \frac{(N-k)}{(k-1)}, \tag{1}$$

where k represents the number of clusters, N is the total number of observations, and SS_B and SS_W are the between-cluster and within-cluster variances, respectively. The equations for SS_B and SS_W are:

$$SS_B = \sum_{i=1}^{k} n_i \left| m_i - m \right|^2, \tag{2}$$

$$SS_W = \sum_{i=1}^{k} \sum_{x \in c_i} \left| x - m_i \right|^2, \tag{3}$$

where n_i is the number of points in the i_{th} cluster with the centroid m_i, m is the total mean of the data, x represents a data point, c_i represents the i_{th} cluster, and $|x - m_i|$ and $|x - m_i|$ are the Euclidean distances (L^2 norm) between the two vectors. The Calinski-Harabasz criterion aims to maximize the variance ratio criterion by identifying the optimal number of clusters with high between-cluster variance and low within-cluster variance.

Davies-Bouldin criterion

We also used the Davies-Bouldin criterion [2] to determine the optimal number of clusters. This criterion captures the ratio of within- and between-cluster distances and is calculated using the following formula:

$$DB = \frac{1}{k} \sum_{i=1}^{k} \max_{j \neq i} D_{i,j}, \tag{4}$$

where $D_{i,j}$ is the ratio of within-to-between cluster distance for clusters i and j, and is defined as:

$$D_{i,j} = \frac{(\bar{d}_i - \bar{d}_j)}{d_{i,j}}, \tag{5}$$

where $\bar{d}_i$ and $\bar{d}_j$ are the average distances between each data point in the i_{th} and j_{th} clusters to their own cluster centroids, and $d_{i,j}$ is the Euclidean distance between the centroids of clusters i and j. The optimal number of clusters is identified by minimizing the Davies-Bouldin index, representing the best within-to-between cluster distance ratio.

Silhouette Analysis

Silhouette Analysis, introduced by [3], is a method to evaluate the quality of clustering by measuring how similar each point is to other points within its cluster compared to points in other clusters. It is defined as follows:

$$S_i = \frac{a_i - b_i}{\max(a_i, b_i)}, \tag{6}$$

Here, a_i represents the average distance between the i_{th} data point and other points in its cluster, and b_i represents the minimum average distance between the i_{th} data point and points in different clusters (minimized across all clusters). A high Silhouette score, ranging between 1 and -1, indicates that the data point is well-clustered within its cluster and poorly matches the data points from other clusters. Conversely, many data points with zero or negative Silhouette values indicate the presence of few or many clusters in the data.

SUPPLEMENTARY INFORMATION (SI) FIGURES

[1] Caliński, T. & Harabasz, J. Communications in Statistics - Theory and Methods. *Communications in Statistics* **3**, 1–27 (1974).

[2] Davies, D. L. & Bouldin, D. W. A Cluster Separation Measure. *IEEE Transactions on Pattern Analysis and Machine Intelligence* **PAMI-1**, 224–227 (1979).

[3] Rousseeuw, P. J. Silhouettes: A graphical aid to the interpretation and validation of cluster analysis. *Journal of Computational and Applied Mathematics* **20**, 53–65 (1987).

[4] Rosch, R. E., Hunter, P. R., Baldeweg, T., Friston, K. J. & Meyer, M. P. Calcium imaging and dynamic causal modelling reveal brain-wide changes in effective connectivity and synaptic dynamics during epileptic seizures. *PLoS computational biology* **14**, e1006375 (2018).

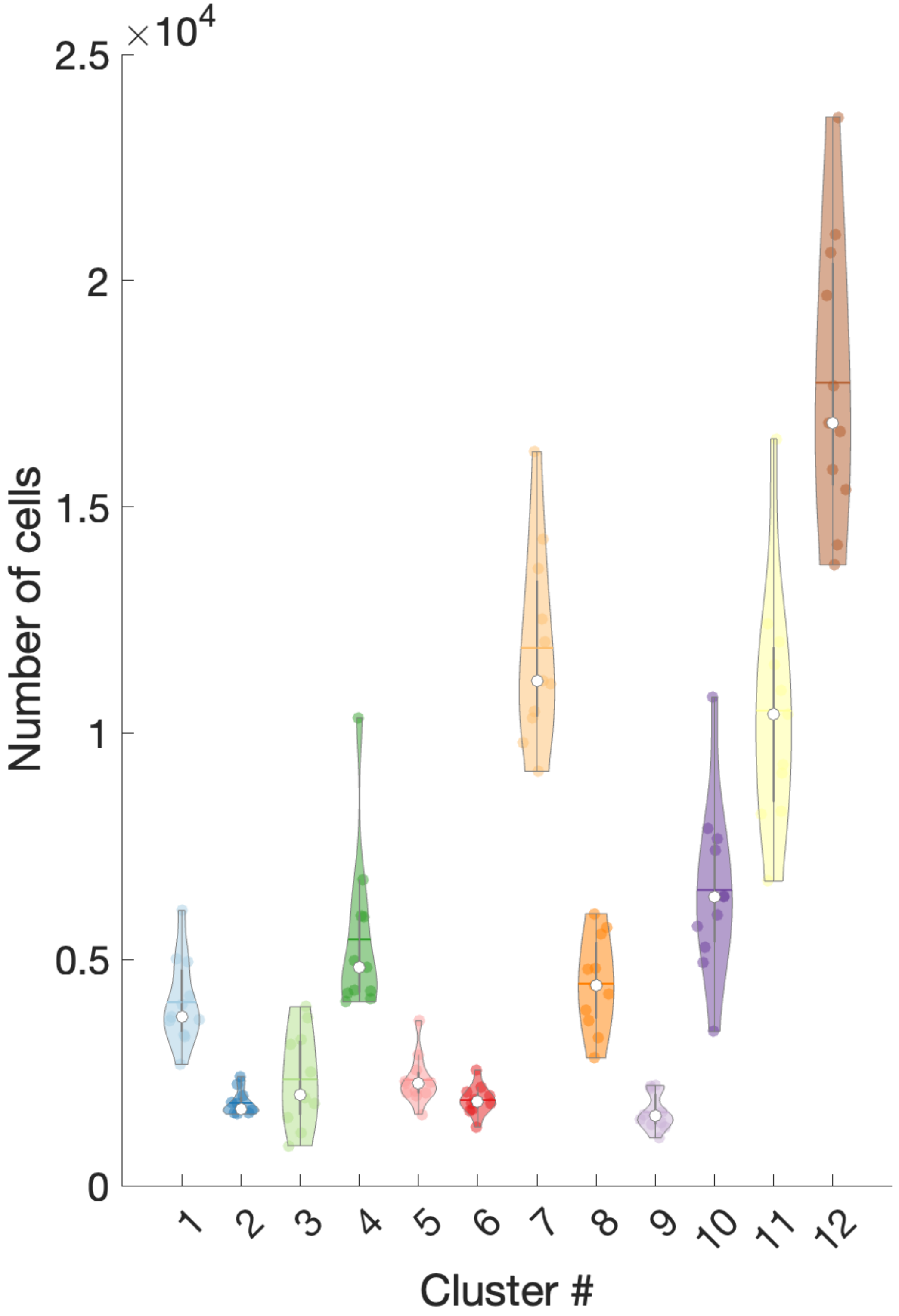

FIG. 1: **Distributions of the number of cells for all 12 functional clusters.** The violin plot displays the distribution of data points representing the number of cells for each zebrafish. The width of the violin at each point represents the density of data at that point, with the broadest part indicating the highest data density. Vertical lines and white dots within each violin represent the distribution's mean and median, respectively.

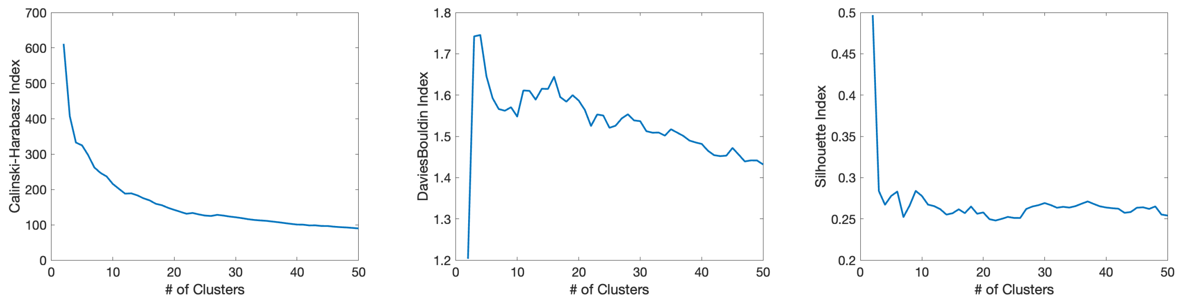

FIG. 2: **Evaluation of functional clusters across different sizes.** The Calinski-Harabasz, Davies-Bouldin, and Silhouette indexes for different $k-$means cluster sizes from left to right plots, respectively. The results show that all three methods identify $N = 2$ as the optimal cluster size with no clear converging results for $N > 2$ clusters across the three methods. See the SI1 section for details about the aforementioned cluster evaluation criteria.

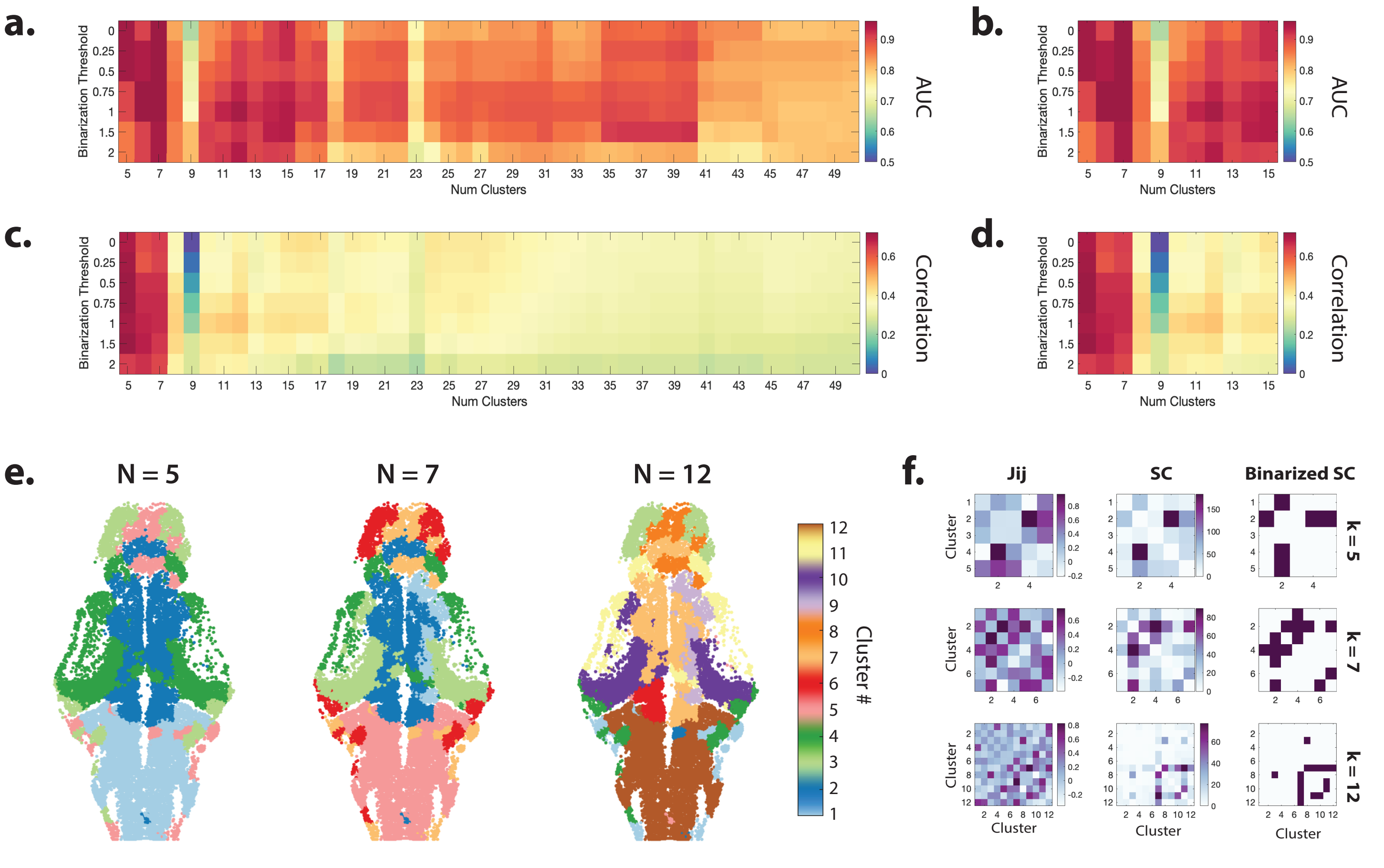

FIG. 3: **Similarity between the Structural and functional connectivity matrices across topological scales. a.** AUC values for detection of thresholded structural edges (> 30% of maximum fiber count) based on J_{ij} matrix estimated using pseudo-likelihood maximization algorithm based on clusters of different sizes and at different binarization thresholds of average cluster activations. **b.** Same as **a**, except the J_{ij} matrix estimated using likelihood maximization algorithm. **c.** The correlation values between the structural edge weights and the J_{ij} weights estimated using the pseudo-likelihood maximization algorithm based on clusters of different sizes and at different binarization thresholds of average cluster activation. **d.** Same as **c**, except the J_{ij} matrix estimated using likelihood maximization algorithm. **e.** Detail of single z-slice demonstrating the spatial location of 5,7, and 12 functional clusters. Note that both $N = 5$ and $N = 7$ clusters identify several spatially disconnected clusters in the anterior-posterior loci. For example, clusters 3 and 5 for $N = 5$ and 6 and 7 for $N = 7$. **f.** The J_{ij} matrices estimated using the likelihood maximization algorithm for high binarization thresholds ($z = 1.5$) for $N =$ 5,7, and 12 clusters. The middle column shows the corresponding structural connectivity (SC) matrices (i.e., fiber count between clusters), and the left column shows the SC matrices thresholded at 30% of maximum SC values.

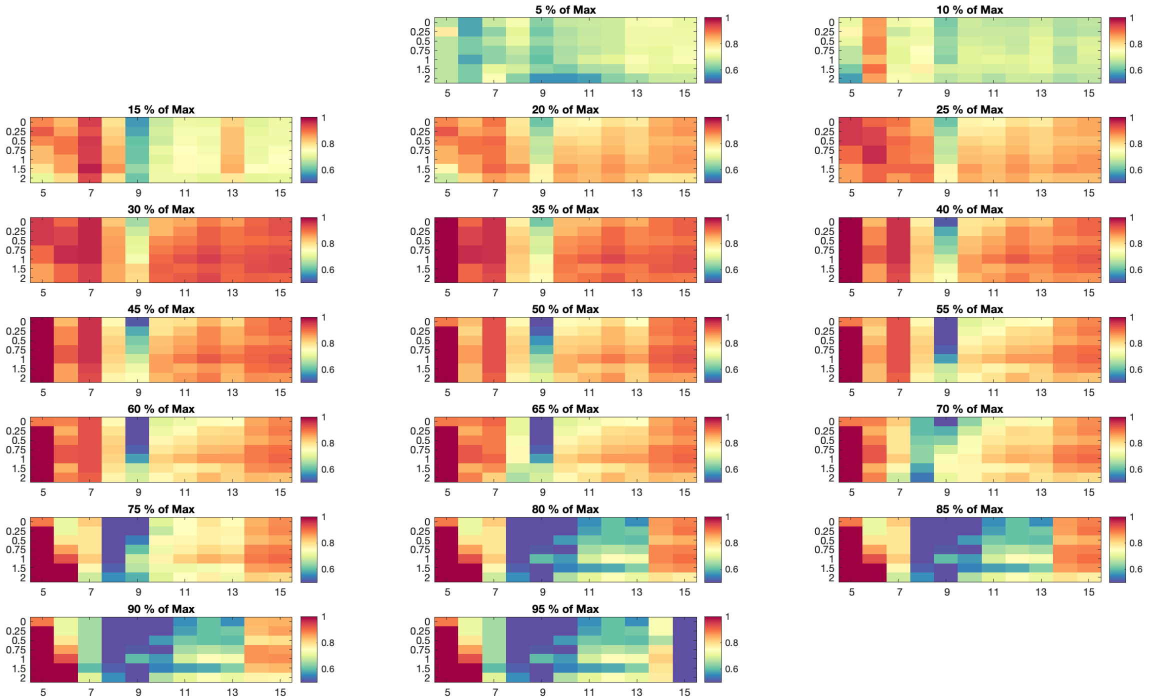

FIG. 4: **Accuracy of detecting structural connections using functional connectivity. a.** AUC values for detection of thresholded structural edges based on J_{ij} matrix estimated using likelihood maximization algorithm based on clusters of different sizes ($N = 5$ to 15) and at different binarization thresholds of average cluster activations. We provided the results for SC matrices thresholded at 5 to 95 % of maximum SC values.

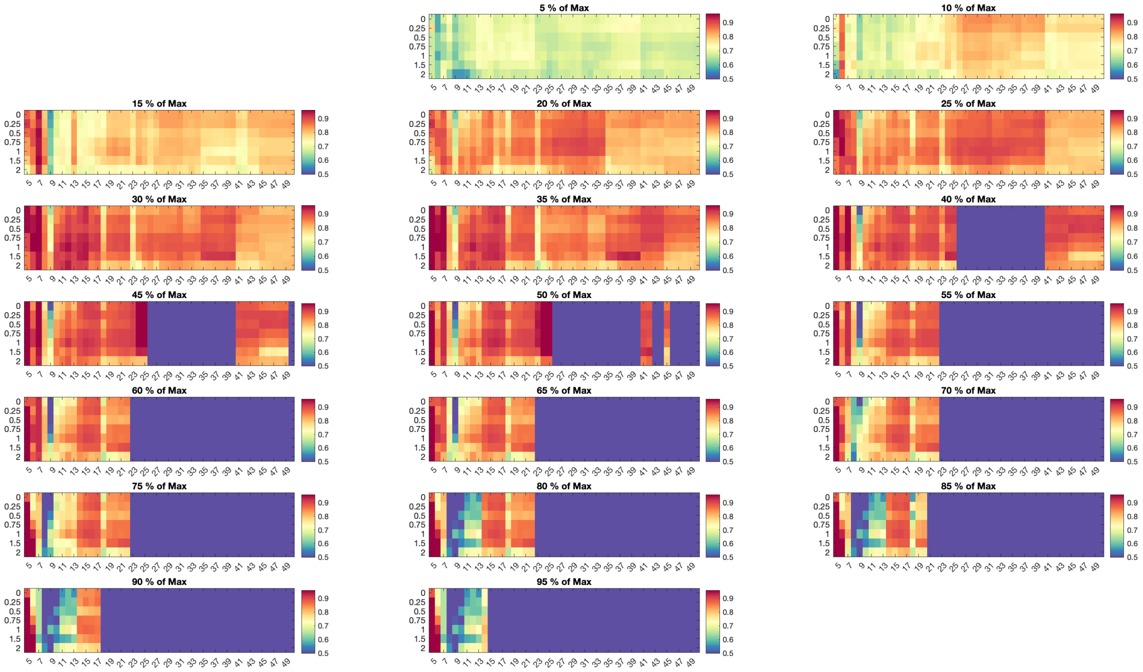

FIG. 5: **Accuracy of detecting structural connections using the functional connectivity estimated using pseudo-likelihood maximization scheme.** **a.** AUC values for detection of thresholded structural edges based on J_{ij} matrix estimated using pseudo-likelihood maximization algorithm based on clusters of different sizes ($N = 5$ to 15) and at different binarization thresholds of average cluster activations. We provided the results for SC matrices thresholded at 5 to 95 % of maximum SC values.

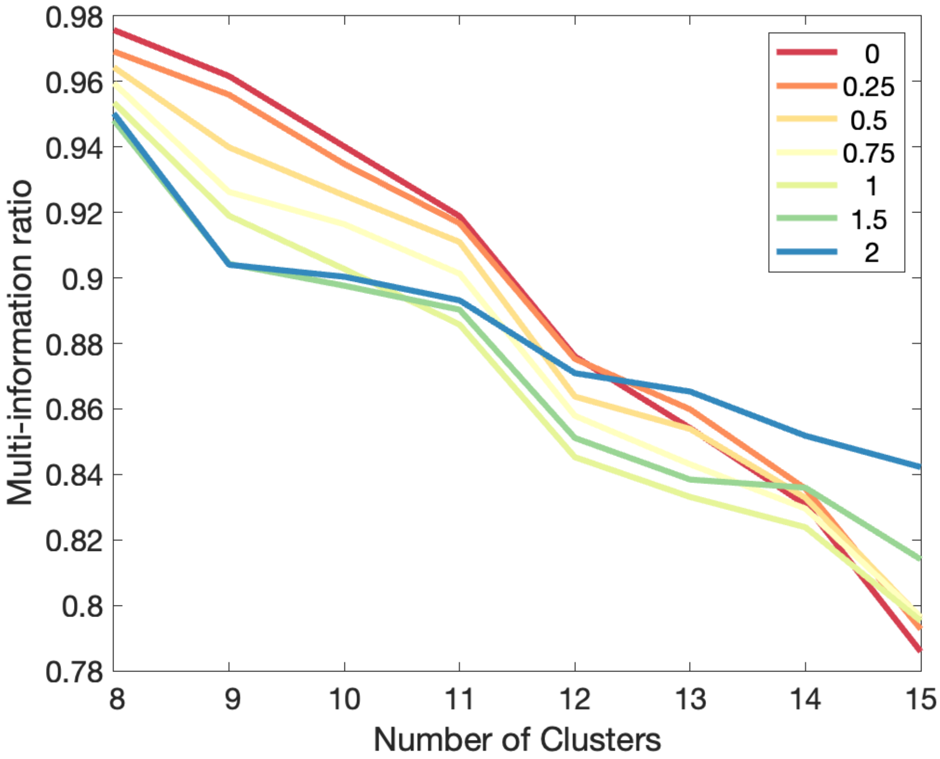

FIG. 6: **Goodness-of-fit of pairwise MEM and the number of clusters.** Here, we show the goodness-of-fit of the pairwise MEM across different cluster sizes and the average cluster activation binarization threshold (color-coded) using the Multi-information ratio. See materials and methods for details on these metrics.

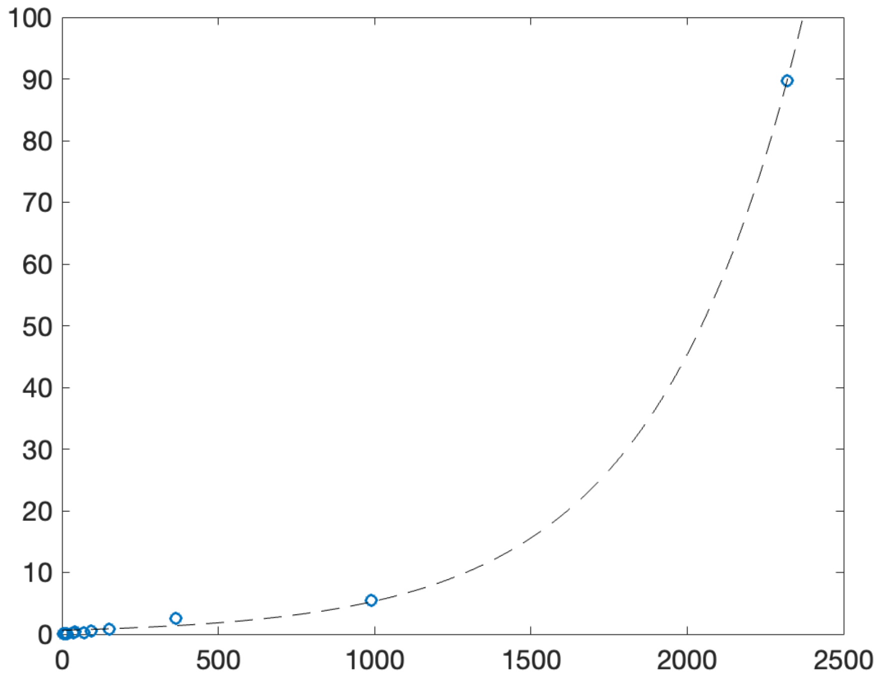

FIG. 7: **Relationship between observed dwell times and the basin size of each attractor state.** The dashed line shows the exponential fit $a \times e^{b \times x}$, Coefficients (with 95% confidence bounds) , $a = 0.6327$ (0.398, 0.8674), $b = 0.002136$ (0.001976, 0.002296)..

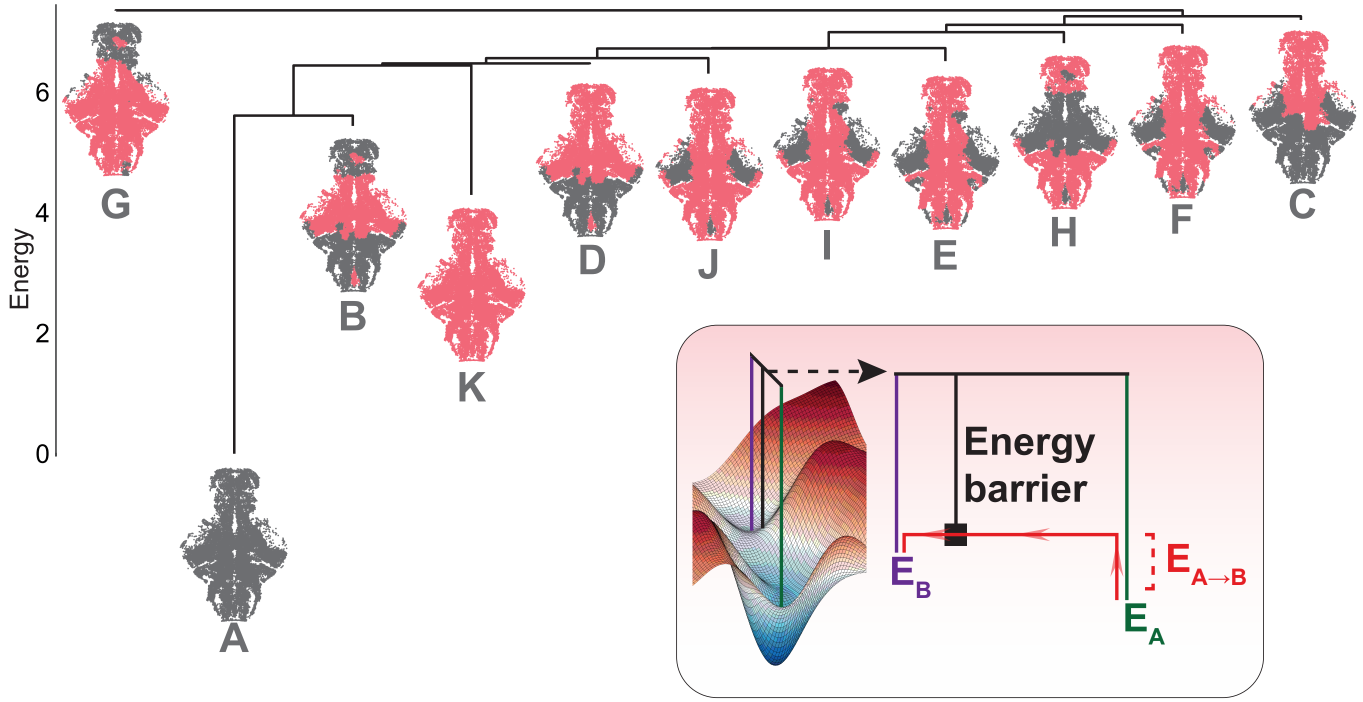

FIG. 8: **Disconnectivity graph between the local minima of the energy landscape. a.** Disconnectivity graph between the local minima of the energy landscape as defined in Fig. ??b. We also illustrate the energy barrier between two example minima (1 and 7), defined by the difference in energy with respect to the saddle point state connecting the two minima.

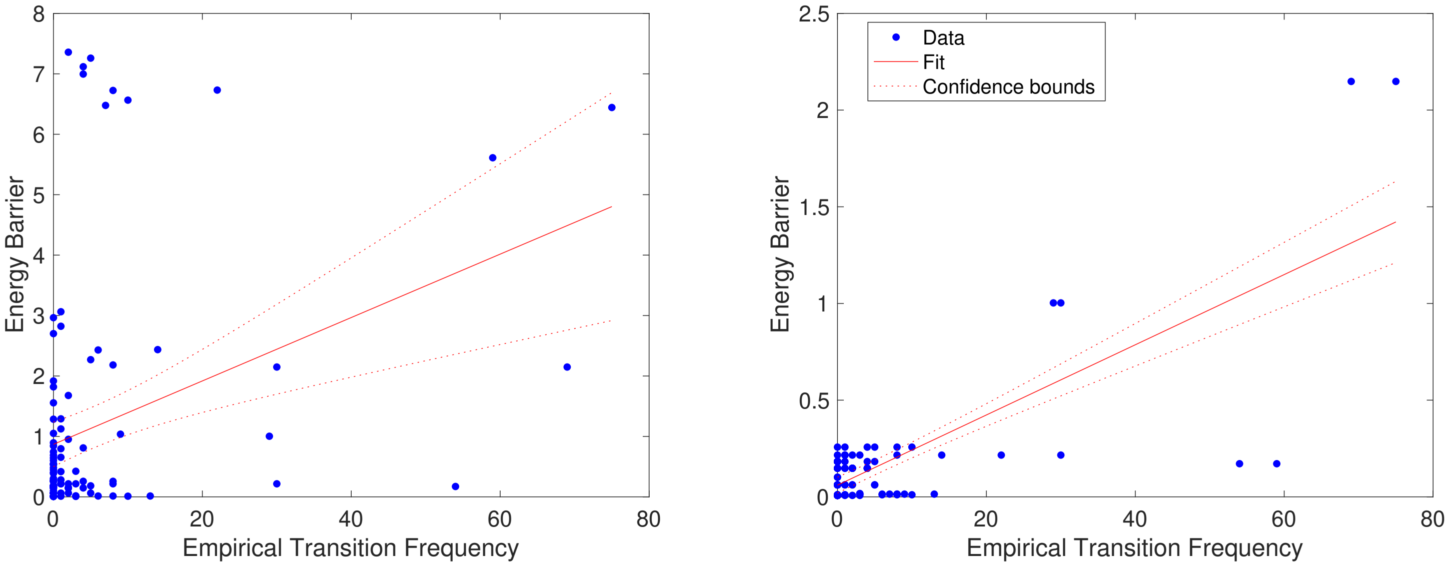

FIG. 9: **The energy barrier between local minima and the transition frequency between them.** (Right) The energy barrier size and the frequency of transitions between the basins of the 11 local minima identified for $N = 12$ clusters. (Left) The same as the right panel except that the y-axis represents the average (i.e., symmetric) energy barrier for each state transition. The solid and dashed red lines show the linear fit (asymmetric barrier fit: p-value $= 8.87 \times 10^{-6}$, $R^2 = 0.12$, symmetric barrier fit: p-value $= 0.004$, $R^2 = 0.57$) and the confidence bounds, respectively.

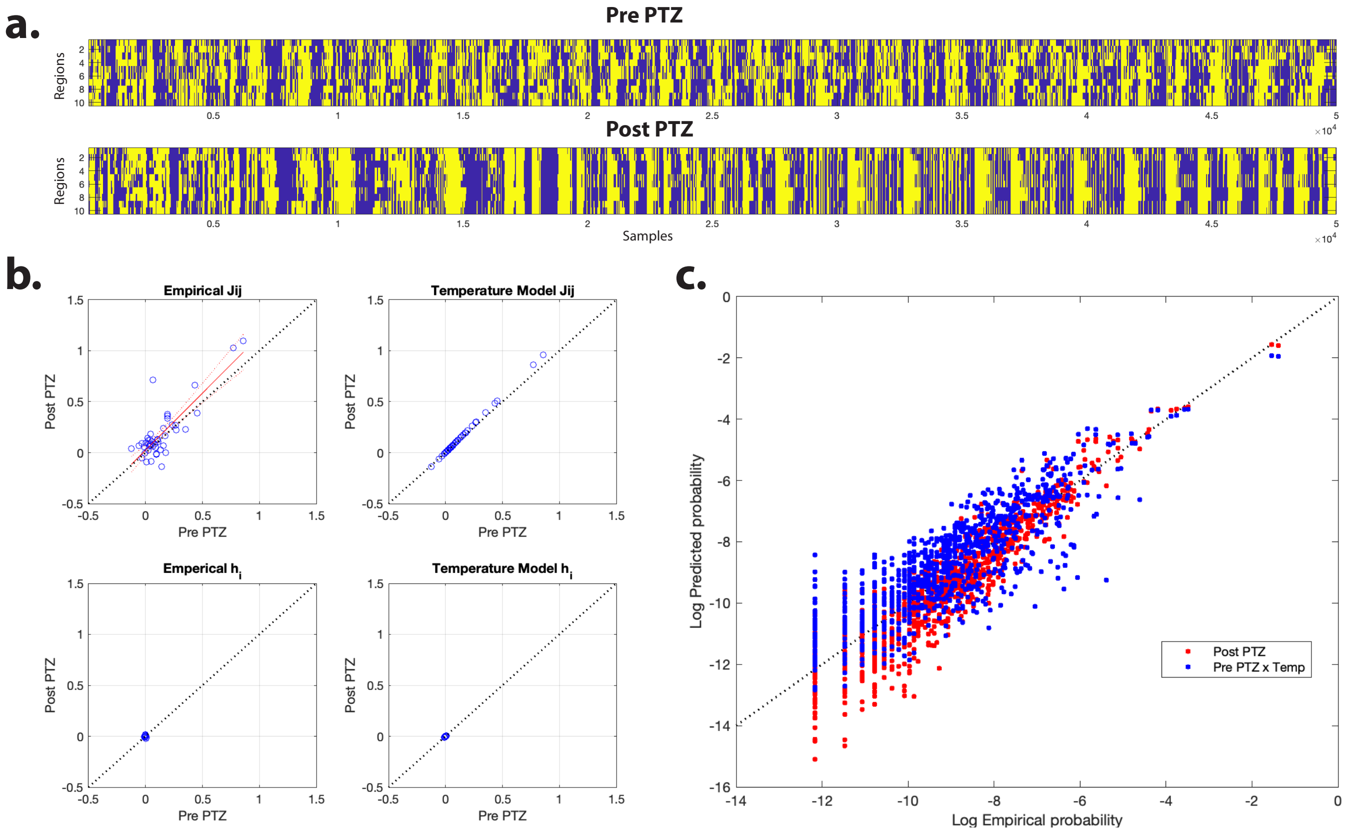

FIG. 10: **Empirical seizure model of zebrafish larvae using Pentylenetetrazol (PTZ).** PTZ is commonly used to induce seizures in zebrafish. Calcium imaging data from three zebrafish larvae, as previously published [4], were utilized in this study. Single plane calcium traces, sampled at 20 Hz, within 10 anatomically defined regions of interest were averaged and detrended using a 100-second moving median. Subsequently, 30-minute pre-PTZ and 54-minute post-PTZ delivery data were collected from each fish and concatenated across three fish separately for pre and post-PTZ. Finally, the concatenated time series were binarized by thresholding at zero. Panel **a** displays 50,000 samples of binarized states for pre (top) and post (bottom) PTZ. **b.** The estimated J_{ij} and h_i parameters of the MEM model from pre- and post-PTZ states are presented on the left. The red lines show the linear fit (slope =1.11, $R^2 = 0.68$, $p-$value $= 3.07 \times 10^-12$) of pre- and post-PTZ J_{ij} values. The plots on the right show the pre-PTS J_{ij} and h_i values against the same values multiplied by the calculated slope in the top left panel, simulating an equivalent global change in the temperature parameters. **C.** The empirical and predicted probability of all post-PTZ states (red), as well as the probability predicted using the aforementioned simulated global temperature change (blue). The post-PTZ pairwise MEM model accounts for 97% of multi-information. The Simulated global temperature change accounts for 91% of multi-information, whereas using the pre-PTZ parameters for predicting the Post-PTZ states only accounts for 88% of multi-information.

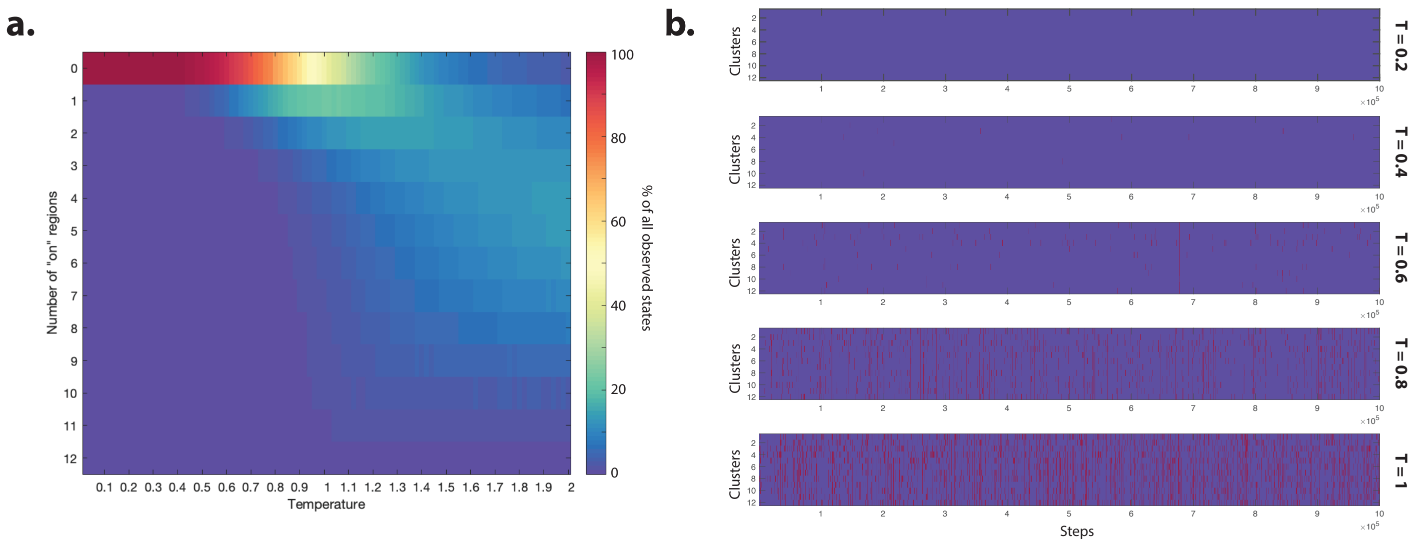

FIG. 11: **Simulated phase transition of the pairwise MEM at higher binarization threshold of cluster activity ($z = 1$). a.** The plot shows the percentage of simulated states with different numbers of "on" clusters at different temperatures, using the Monte Carlo Markov Chain (MCMC) simulation method. **b.** MCMC-simulated state transitions at five different sample temperatures. The "on" clusters are highlighted in red. Note that in the Ising model of the ferromagnetism, the spins' direction in the absence of an external magnetic field (i.e., $h = 0$) is randomly assigned, the phase transition to ferromagnetic is symmetric as the net magnetic moment can point to either direction. However, the presence of an external magnetic field (i.e., $h \neq 0$) can lead to symmetry breaking. For instance, our simulations show that the overall negative distribution of the estimated h (i.e., clusters' activation propensity) promotes transition to the silent state following cooling (i.e., increased global connectivity) at higher cluster activation binarization thresholds (e.g., $z = 1$). However, the brain-wide active state's duration depends on the average cluster activation time series binarization threshold. Lowering the binarization thresholds results in more prolonged global active and seizure-like states at lower temperatures (e.g., $z = 0$ in manuscript Fig. 4).

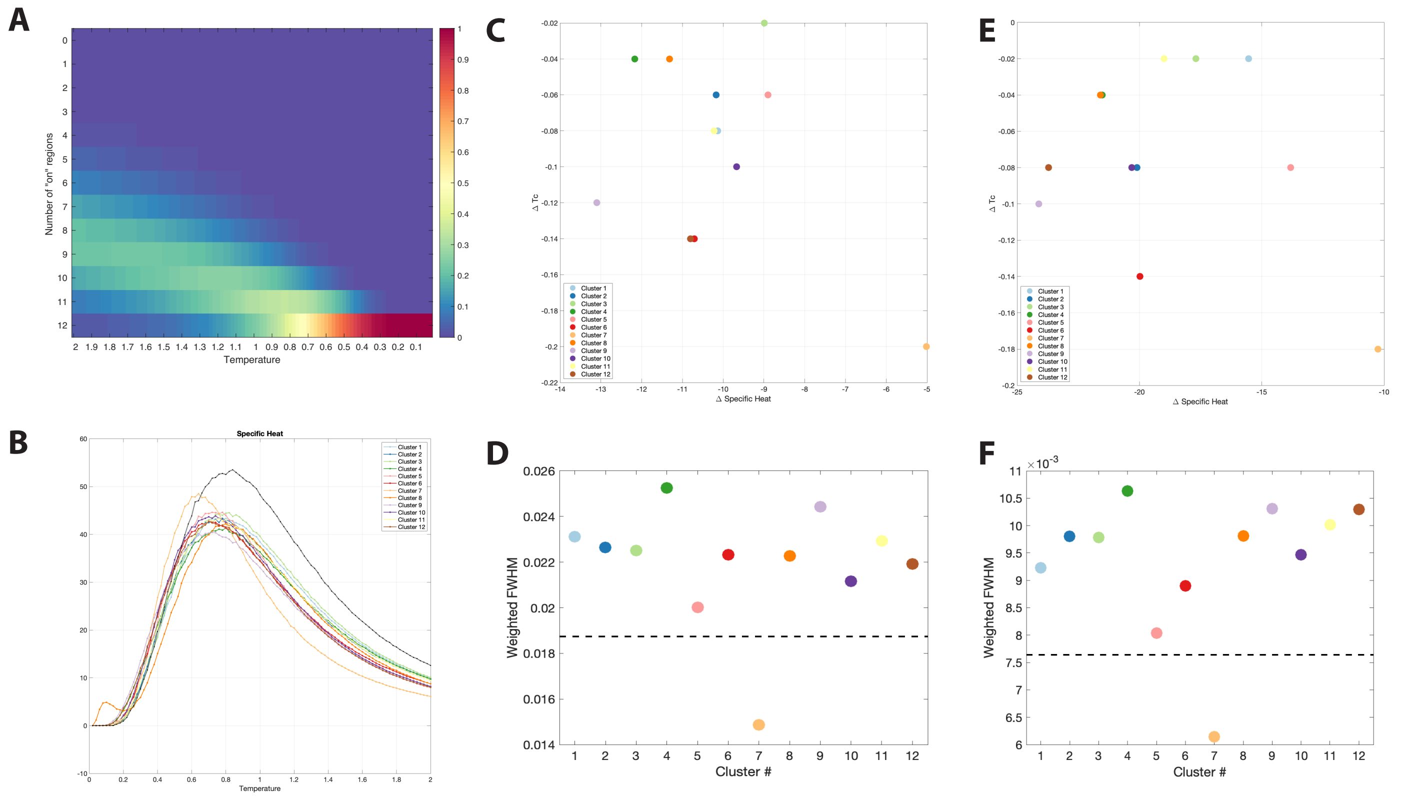

FIG. 12: **Transitions of global activity in the pairwise MEM for (-1, 1) state definition. a.** Proportion of simulated states with different numbers of active clusters at different temperatures, simulated using the MCMC algorithm, for (-1, 1) state definition **b.** The specific heat curves before (black) and after (color-coded for each cluster) virtual resection. **c.** the change in the peak of the curves following the resections. **d.** Full width at half maximum (FWHM) values of the specific heat curves. Panels E and F show the same results as panels C and D for (0,1) state definition.

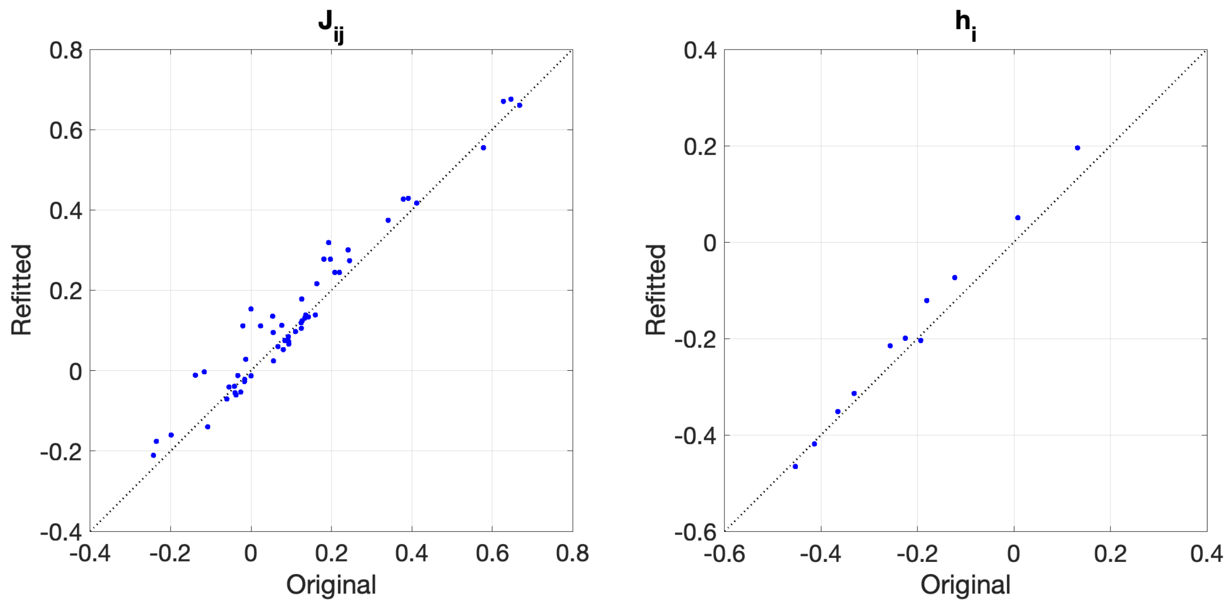

FIG. 13: **Comparison between the estimated J_{ij} and hi parameters after ignoring cluster 7** ($N = 11$) **and the corresponding J_{ij} and h_i parameters from the full system** ($N = 12$)**.** Note that refitting the model after ignoring the high degree cluster 7 results in an artificial increase in many J_{ij} elements.

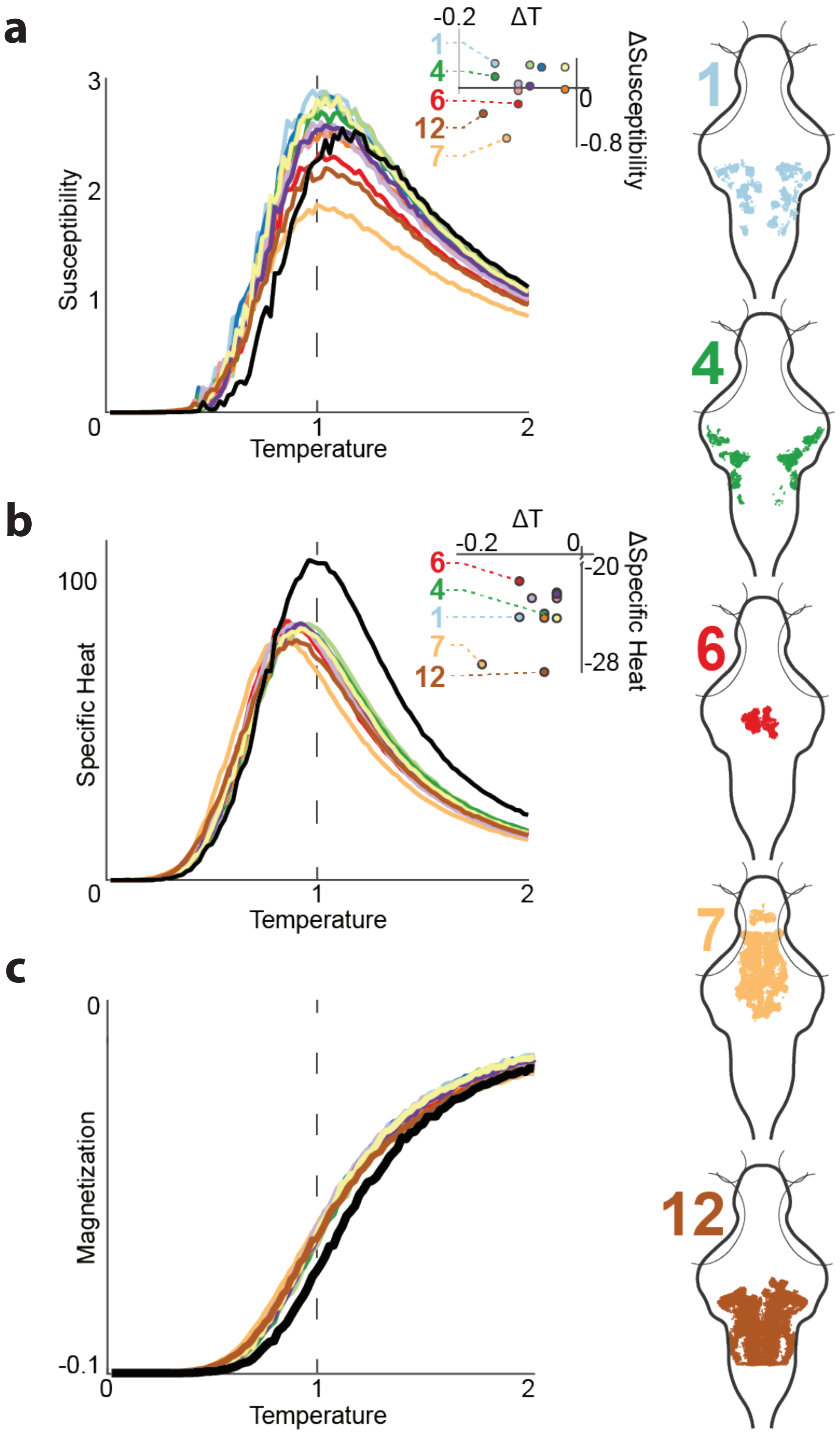

FIG. 14: **transitions of pairwise MEM of the zebrafish brain at higher binarization threshold of cluster activity ($z = 1$). a.** The susceptibility, **b.** specific heat **c.** and magnetization curves before (black) and after (color-coded for each cluster) virtual resection. The insets show the change in the peak of the curves following the resections.
